# Phosphatidic Acid‐TRIM59‐Olig2 Signaling Couples Metabolic Dysfunction to Myelination Failure in PWMI

**DOI:** 10.1002/advs.202521296

**Published:** 2026-02-18

**Authors:** Xinyu Li, Yanan Liu, Meng Zhang, Yiwei Liu, Meiting Liu, Kaishen Zhu, Zhian Liu, Chao Ren, Ruiqin Yao

**Affiliations:** ^1^ Department of Cell Biology and Neurobiology Xuzhou Key Laboratory of Neurobiology Xuzhou Medical University Xuzhou Jiangsu Province China; ^2^ Department of Neurology Affiliated Hospital of Xuzhou Medical University Xuzhou Jiangsu Province China; ^3^ Department of Human Anatomy Xuzhou Medical University Xuzhou Jiangsu Province China; ^4^ Department of Urology Affiliated Hospital of Xuzhou Medical University Xuzhou Jiangsu Province China; ^5^ Department of Neurology Yantai Yuhuangding Hospital Qingdao University Yantai Shandong Province China; ^6^ Shandong Provincial Key Laboratory of Neuroimmune Interaction and Regulation Yantai Yuhuangding Hospital Qingdao University Yantai Shandong Province China; ^7^ Yantai Municipal Key Medical and Health Laboratory of Yantai Yuhuangding Hospital (Interdisciplinary Brain Science and Geriatric Health Laboratory), Yantai Yuhuangding Hospital Qingdao University Yantai Shandong Province China

**Keywords:** cerebral palsy, OPCs, phosphatidic acid, PWMI, TRIM59

## Abstract

Cerebral palsy (CP), mainly resulting from preterm white matter injury (PWMI), remains a leading neurodevelopmental disorder. While oligodendrocyte precursor cell (OPC) differentiation failure is central to PWMI pathology, the metabolic mechanisms remain unclear. Here, untargeted lipidomic and metabolomic profiling of serum samples from retrospective and prospective cohorts of preterm infants identified a CP‐associated metabolic signature, highlighting phosphatidic acid (PA) as a top candidate that was consistently elevated and showed strong discriminative potential. Increased PA levels were validated in both serum and brains of PWMI mice and in OPCs subjected to oxygen‐glucose deprivation/reoxygenation (OGD/R), where PA impaired OPC differentiation and myelination. Mechanistically, PA interacted with and stabilized the E3 ubiquitin ligase TRIM59, increasing its protein abundance and half‐life without affecting mRNA levels. Elevated TRIM59 promoted proteasomal degradation of the oligodendrocyte lineage transcription factor Olig2, a key regulator of OPC maturation. Inhibition of PA synthesis restored Olig2 expression, improved myelination, and rescued differentiation deficits in PWMI mice. Collectively, this study identifies PA as a potential metabolic risk factor associated with preterm CP and uncovers a PA‐TRIM59‐Olig2 signaling axis linking lipid metabolism to OPC differentiation failure and PWMI.

## Graphical abstract

PA accumulates after hypoxic‐ischemic injury and stabilizes the E3 ligase TRIM59 in OPCs. Stabilized TRIM59 enhances ubiquitination and degradation of Olig2, blocking differentiation and causing hypomyelination in PWMI. Modulating PA synthesis restores Olig2 levels, improves myelination, and ameliorates behavioral deficits, defining a metabolically driven pathway linking lipid dysregulation to cerebral palsy‐related pathology.

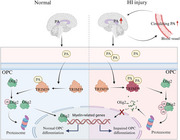

## 1 Introduction

Cerebral palsy (CP) is the most common motor disability in childhood, with over one‐third of cases linked to prematurity and the highest risk in infants born before 28 weeks of gestation [1, 2]. Preterm CP is most frequently associated with preterm white matter injury (PWMI), a pathology strongly linked to hypoxic‐ischemic (HI) insults during early brain development [3, 4]. PWMI, characterized by arrested oligodendrocyte precursor cell (OPC) differentiation and impaired myelination, represents the predominant neuropathological substrate in this vulnerable population [3, 5, 6]. Despite advances in neonatal care, early diagnosis and effective intervention remain limited [7, 8], largely due to the lack of reliable biomarkers and incomplete understanding of the underlying mechanisms.

Lipid metabolism plays a pivotal role in OPC differentiation. Beyond their structural functions, lipids act as signaling molecules and regulators of protein localization and post‐translational modification during oligodendrocyte lineage progression [9, 10]. Given this metabolic vulnerability, metabolomic and lipidomic profiling offer a promising strategy to identify predictive biomarkers and mechanistic drivers of CP [11, 12]. Compared to genetic or epigenetic analyses, metabolites, being direct products of cellular processes, can more accurately capture early pathological changes [11].

To address this need, we performed untargeted serum metabolomic and lipidomic profiling in retrospective and prospective cohorts of preterm infants. Integrative bioinformatics and machine learning identified a discriminative metabolite signature, with phosphatidic acid (PA), a central intermediate in glycerophospholipid metabolism, emerging as a top candidate. PA was consistently elevated in infants who later developed CP and showed a robust association with CP status across cohorts. Increased PA levels were further validated in serum and brain tissues of PWMI mouse models and in OPCs subjected to oxygen–glucose deprivation/reoxygenation (OGD/R), underscoring its clinical and biological relevance.

Despite structural simplicity, PA exhibits high biosynthetic turnover in the central nervous system (CNS) and functions as a multifunctional signaling lipid that modulates membrane curvature, protein interactions, and intracellular signaling [13, 14]. Notably, PA has been implicated in neurodegenerative disorders by binding α‐synuclein, promoting pathological aggregation, enhancing amyloid‐β accumulation, and impairing Schwann cell differentiation [15, 16, 17, 18]. Proteomic profiling identified tripartite motif‐containing protein 59 (TRIM59), an E3 ubiquitin ligase highly expressed in oligodendrocytes, as a potential PA‐binding protein [19], with molecular docking predicting interaction near Lys114, suggesting a mechanism for PA‐mediated stabilization. Previous studies have implicated TRIM59 in diverse biological processes, including regulation of cell cycle progression, modulation of inflammatory signaling, and promotion of tumor growth and metastasis through ubiquitin‐dependent control of key signaling proteins [20]. Although TRIM59 regulates protein ubiquitination in various contexts, its role in OPC lineage specification remains unknown.

Building on our clinical findings implicating PA as a candidate biomarker associated with CP, we investigated the mechanisms underlying its pathogenic role. We demonstrate that HI injury‐induced PA accumulation stabilizes TRIM59, leading to ubiquitin‐dependent degradation of the transcription factor Olig2, a key regulator of oligodendrocyte lineage progression. This newly defined PA‐TRIM59‐Olig2 axis establishes a lipid‐E3 ligase‐transcription factor cascade linking metabolic dysregulation to OPC maturation failure and impaired myelination. These findings fill a critical gap in understanding PWMI pathogenesis and highlight lipid‐protein interactions as potential therapeutic targets in preterm brain injury.

## 2 Materials and Methods

### 2.1 Clinical Sample Collection and Study Design

We conducted retrospective and prospective studies at Xuzhou Medical University. The retrospective cohort included 30 children (1‐4 years) with CP and 30 matched preterm controls, while the prospective cohort enrolled 30 preterm infants later diagnosed with CP and 30 controls from the Neonatal Intensive Care Unit (NICU). Diagnosis followed international and Chinese criteria, with exclusion of genetic/metabolic disorders, malformations, or severe systemic illness. Sex was recorded for all participants and did not differ significantly between CP and control groups. CP severity was assessed using GMFCS. Peripheral venous blood (1‐3 mL) was collected, processed into serum, and stored at ‐80°C. The study was approved by the Ethical Committee of the Affiliated Hospital of Xuzhou Medical University (Approval No. XYFY2023‐KL165‐01), and written informed consent was obtained from all participants’ legal guardians. Detailed procedures are provided in the Supplementary Methods.

### 2.2 Metabolomic and Lipidomic Profiling

Serum metabolites and lipids were extracted using standard methanol (041467‐AK, Thermo Fisher Scientific)/acetonitrile (A9541, Thermo Fisher Scientific) and MTBE (B802535, Adamas‐beta, Shanghai, China)‐based protocols, and analyzed on a Thermo UHPLC‐Q Exactive system. Data were processed using Progenesis QI and LipidSearch. To ensure data quality and reproducibility, pooled quality control (QC) samples were generated by combining equal aliquots from each serum sample and were injected periodically throughout the analytical run to monitor instrument stability and signal drift. Features with a relative standard deviation (RSD) >30% in QC samples were excluded from downstream analyses. Signal intensities were normalized to total ion current (TIC) to correct for inter‐sample variability, followed by log transformation and Pareto scaling prior to multivariate analyses. Missing values were imputed using a k‐nearest neighbor algorithm when appropriate.

Differential metabolites/lipids were identified by Variable Importance in Projection (VIP) >1 and *P* < 0.05. Pathway enrichment was performed with KEGG, and diagnostic models were evaluated using support vector machine (SVM) and receiver operating characteristic (ROC) analysis. Detailed procedures are provided in the Supplementary Methods.

### 2.3 Animals Treatments and Ethical Approval

Specific pathogen‐free (SPF)‐grade C57BL/6J mice were obtained from the Animal Center of Xuzhou Medical University. Mice were housed in individually ventilated cages under a 12 h light/dark cycle with controlled temperature (20‐22°C) and humidity (50%–60%), with free access to chow and water. Breeding was initiated at puberty (males, 8 weeks; females, 6 weeks). The presence of a vaginal plug was used to determine pregnancy, and the day of birth was designated postnatal day 0 (P0). All procedures were approved by the Ethics Committee of Experimental Animal Center of Xuzhou Medical University (approval No. SYXK2005‐0018) and conducted in accordance with the NIH Guide for the Care and Use of Laboratory Animals. **PA treatment**: P7 pups were randomly assigned into three groups (n = 6 each): PBS, PA (Sigma, Missouri, USA) 1.6 mg/kg, and PA 3.2 mg/kg. Intraperitoneal injections were administered daily at 9:00 a.m. for 7 consecutive days, and brains were harvested at P14 for subsequent analyses. **FIPI treatment**: Beginning at P3, pups received daily intraperitoneal injections of the Phospholipase D (PLD) inhibitor FIPI (5‐fluoro‐2‐indolyl des‐chlorohalopemide, 3 mg/kg, dissolved in 4% DMSO/96% saline; MCE, New Jersey, USA) for 8 consecutive days before tissue collection.

### 2.4 Induction of PWMI Model

To model the HI conditions commonly implicated in PWMI, neonatal mice were subjected to HI injury as described below. On postnatal day 3 (P3), unsexed pups were anesthetized with isoflurane (3% induction, 1.5% maintenance) and placed on a thermostatically controlled table at 37°C. Under a stereomicroscope, the right common carotid artery was isolated and ligated with 6‐0 surgical silk, after which the incision was closed. Sham‐operated pups underwent the same procedure without ligation. The surgery was completed within 5 min. Immediately afterward, pups were exposed to a hypoxic environment (8% O_2_/92% N_2_) for 1.5 h in a chamber with real‐time gas monitoring (ProOx 110, BioSpherix, USA). Following hypoxia, pups were returned to their dams and maintained under standard housing conditions until sacrifice.

### 2.5 Measurement of Phosphatidic Acid (PA)

PA levels were determined using the PicoProbe Phosphatidic Acid Assay Kit (ab273335, Abcam, Cambridge, UK) following the manufacturer's instructions. For tissue samples, brains were homogenized in the supplied assay buffer, and lipids were extracted according to the kit protocol. For cultured cells, cells were washed 2–3 times with PBS, counted, and resuspended in PA assay buffer (1 mL per 1 × 10^6^ cells). Cells were collected using a cell scraper, transferred to glass tubes, and vortexed for 5 min. In both cases, extracted lipids were solubilized in 5% Triton X‐100. Parallel “sample” and “background control” wells were prepared, and a standard curve was generated using serial dilutions of the PA standard. The converter mix was added only to the sample and standard wells, incubated at 45°C for 1 h, followed by the addition of the reaction mix and incubation at 37°C for 30 min. Fluorescence (Ex/Em = 535/587 nm) was measured with a microplate reader, and PA concentrations were calculated from the standard curve and normalized to protein content.

### 2.6 Primary OPC Culture and OGD Assay

Primary OPCs were isolated from neonatal cortices and purified by differential shaking. Differentiation was induced by triiodothyronine (T3) (GLPBIO, Montclair, USA) withdrawal of mitogens. For ischemia modeling, cells were subjected to OGD (glucose‐free DMEM, 1% O_2_, 3, 6, or 9 h) followed by 24 h reoxygenation. Detailed procedures are provided in the Supplementary Methods.

### 2.7 Molecular and Cellular Assays

Immunofluorescence (IF) and Western blotting (WB) were performed using standard protocols with antibodies against MBP, PDGFR‐α, Olig proteins, TRIM59, SOX10, Sip1, NKX2.2, and controls. TRIM59 knockdown was achieved by siRNA transfection (sequences in Supplementary Table 1). Detailed antibody information and complete experimental procedures are provided in the Supplementary Methods.

### 2.8 Cell Viability Assay

Purified OPCs were seeded at 2 × 10^4^ cells/well in 96‐well plates and allowed to attach for 24 h. Cells were then treated with PA or PA synthesis inhibitors for the indicated durations. Cell viability was measured using CCK‐8 according to the manufacturer's instructions, and absorbance was read at 450 nm.

### 2.9 Co‐Immunoprecipitation (Co‐IP) and Ubiquitination Assays

Cells were lysed in ice‐cold IP lysis buffer (P0013, Beyotime, Shanghai, China) supplemented with a 1:100 dilution of Protease Inhibitor Cocktail (P1005, Beyotime, Shanghai, China). Lysates were incubated on ice for 30 min with gentle agitation and vortexed intermittently to ensure complete cell disruption. The lysates were centrifuged at 13,000 rpm for 20 min at 4°C, and the supernatants were collected. For input controls, aliquots of lysates were mixed with SDS loading buffer and boiled for 10 min.

For immunoprecipitation, 20 µL of the indicated antibodies (e.g., anti‐Olig2, anti‐TRIM59) pre‐bound to Protein A/G beads were added to the cleared lysates and rotated overnight at 4°C. The beads were washed three times with lysis buffer (5 min each) to remove nonspecific binding and subsequently eluted by boiling in SDS loading buffer for 10 min. Eluates and input samples were resolved by SDS‐PAGE and analyzed by WB using the indicated antibodies.

For ubiquitination assays, cells were lysed under denaturing conditions (1% SDS, 95°C, 10 min) to disrupt non‐covalent protein‐protein interactions, followed by 10‐fold dilution with ice‐cold IP buffer to reduce SDS concentration before immunoprecipitation. Ubiquitinated proteins were detected by WB using an anti‐ubiquitin antibody after immunoprecipitation with anti‐Olig2 or anti‐TRIM59 antibodies. Relative ubiquitination levels were quantified based on the intensity of high–molecular weight smears normalized to input controls.

### 2.10 Reverse Transcription and Quantitative PCR (RT‐qPCR)

RNA was extracted with TRIzol (15596018, Invitrogen, State of California, USA), reverse‐transcribed, and quantified by SYBR Green‐based qPCR. GAPDH served as an internal control. The primer sequences used for qPCR are listed in Supplementary Table 2.

### 2.11 Liposome Flotation Assay

Purified TRIM59 protein (Figure S1) (1 µM) was incubated with liposomes containing Phosphatidylcholine (PC), PA, and Cardiolipin (CL) (300 µM) at 37°C for 20 min. Samples were mixed with 30% sucrose at the bottom of an ultracentrifuge tube, overlaid with 25% sucrose and buffer, and centrifuged at 240 000 × g for 2 h at 4°C. Fractions were collected and analyzed by western blot using anti‐TRIM59 antibody to assess protein‐lipid binding. Detailed procedures are provided in the Supplementary Methods.

### 2.12 Behavioral Tests

#### 2.12.1 Morris Water Maze (MWM)

Spatial learning and memory were evaluated in a circular pool (120 cm diameter) with a submerged platform (8 cm). Mice underwent four training trials per day for 4 days, followed by a probe trial on day 5. Swimming paths and performance indices were recorded using ANY‐maze (Stoelting, USA)

#### 2.12.2 Open Field Test

Locomotor activity and anxiety‐like behavior were assessed in a 50 × 50 × 30 cm arena for 5 min. Distance traveled, mean speed, and time spent in central versus peripheral zones were analyzed.

#### 2.12.3 Y‐Maze

Short‐term spatial memory was tested using a two‐trial protocol (training with one arm blocked, followed by a test session 4 h later with all arms open). Novel arm entries and exploration time were quantified.

#### 2.12.4 Rotarod

Motor coordination was measured on an accelerating rotarod. Latency to fall (cut‐off 300 s) was averaged across three trials. Detailed procedures are provided in the Supplementary Methods.

### 2.13 Transmission Electron Microscopy (TEM)

Mice were perfused with electron microscopy fixative, and the corpus callosum was rapidly dissected on ice. Uniform tissue blocks (∼1 mm^3^) were fixed, post‐fixed with osmium tetroxide, dehydrated through graded ethanol and acetone, and embedded in epoxy resin. Ultrathin sections (∼50 nm) were prepared, stained with lead citrate, and examined by transmission electron microscopy. Images were acquired at 6000× magnification. Myelin ultrastructure was quantified by calculating the G‐ratio using ImageJ. Three biological replicates were analyzed per group.

### 2.14 Statistics

All statistical analyses are described here. Data are presented as mean ± SD unless otherwise indicated. Normality and homogeneity of variance were assessed by the Shapiro‐Wilk and Levene tests, respectively. For two‐group comparisons, unpaired two‐tailed Student's t‐tests were used for normally distributed data, and Mann‐Whitney U tests for non‐normal data. For comparisons among three or more groups, one‐way ANOVA was applied, followed by Dunnett's or LSD post hoc tests as appropriate; two‐way ANOVA was used when two independent factors were present. ROC curves and area under the curve (AUC) were used to evaluate diagnostic models; SVM was applied for classification model building. For SVM modeling, metabolite features were first selected using LASSO regression to reduce dimensionality. The resulting feature set was used to train an SVM classifier. Model performance was evaluated by internal cross‐validation, and discriminative ability was assessed using ROC curve analysis. Sample size for human cohorts was determined based on study design (30 cases and 30 controls per cohort); sample sizes for animal and cellular experiments were chosen based on previous literature and pilot data to provide adequate power. No data points were excluded. Randomization was used for the allocation of animals to treatment groups and for the assignment of wells in cell assays. Investigators were blinded to group allocation during data acquisition and analysis wherever feasible. Statistical tests were two‐sided, and *p* < 0.05 was considered significant. All analyses were performed using GraphPad Prism 9.0; details of specific tests applied to each dataset are provided in the figure legends and Supplementary Methods.

## 3 Results

### 3.1 Dysregulated Metabolites and Candidate Biomarkers in Preterm CP

We first performed untargeted serum metabolomic and lipidomic profiling in 30 preterm infants with CP and 30 matched preterm controls. Demographic analysis revealed significant group differences, including lower gestational age and a higher incidence of perinatal complications in the CP cohort. Postnatal Magnetic Resonance Imaging (MRI) confirmed that white matter abnormalities were strongly associated with CP (Table 1).

**TABLE 1:** Analysis of clinical characteristics of the retrospective cohort.

Clinical characteristics	CP group	Control group	*p* value
Demographic			
Male, n (%)	21 (70%)	17 (56.7%)	0.284
Gestational age at birth, wk, mean (SE)	33.5 (0.53)	34.8 (0.48)	0.012^*^
Birth weight, g, mean (SE)	2496 (126)	2730 (135)	0.269
Current age, m, mean (SE)	24.2 (2.22)	23.7 (2.41)	0.559
Maternal			
Maternal age, y, mean (SE)	29.6 (0.56)	28.8 (0.47)	0.302
Placenta previa, n (%)	6 (20%)	5 (16.7%)	0.739
Preeclampsia, n (%)	8 (26.7%)	2 (6.7%)	0.038^*^
Primigravida, n (%)	10 (33.3%)	11 (36.7%)	0.787
Antenatal magnesium sulphate, n (%)	9 (30%)	8 (26.7%)	0.774
Other accompanying diseases, n (%)	7 (23.3%)	8 (26.7%)	0.766
Perinatal			
Placental abruption, n (%)	5 (16.7%)	3 (10%)	0.706
Chorioamnionitis, n (%)	7 (23.3%)	1 (3.3%)	0.052
Cesarean delivery, n (%)	16 (53.3%)	14 (46.7%)	0.606
1‐min Apgar score, mean (SE)	4.6 (0.49)	6.1 (0.36)	0.036^*^
5‐min Apgar score, mean (SE)	6.6 (0.50)	8.4 (0.35)	0.007^**^
Fetal growth restriction, n (%)	8 (26.7%)	2 (6.7%)	0.038^*^
Kernicterus, n (%)	14 (46.7%)	7 (23.3%)	0.058
Postnatal			
Hypotension, n (%)	13 (43.3%)	3 (10%)	0.004^**^
Infection, n (%)	15 (50%)	3 (10%)	0.001^**^
Mechanical ventilation≥ 7d, n (%)	14 (46.7%)	5 (16.7%)	0.012^*^
Chronic lung disease, n (%)	8 (26.7%)	3 (10%)	0.095
postnatal corticosteroids, n (%)	11 (36.7%)	4 (13.3%)	0.037^*^
Imaging			
Postnatal age at MRI, wk, mean (SE)	5.8 (0.29)	5.8 (0.27)	0.945
White matter abnormalities, n (%)	18 (60%)	6 (20%)	0.002^**^
Cerebrospinal fluid space abnormalities, n (%)	9 (30%)	5 (17%)	0.222
Cerebellar abnormalities, n (%)	3 (1%)	0	0.237

*Note*: The chi‐square test was employed to compare the counting data in groups. For comparison of measurement data between groups, Student's t test was used for normality, and the Mann‐Whitney U test was used for non‐normality. The statistically significant *p* values are highlighted in red.

**FIGURE 1:**
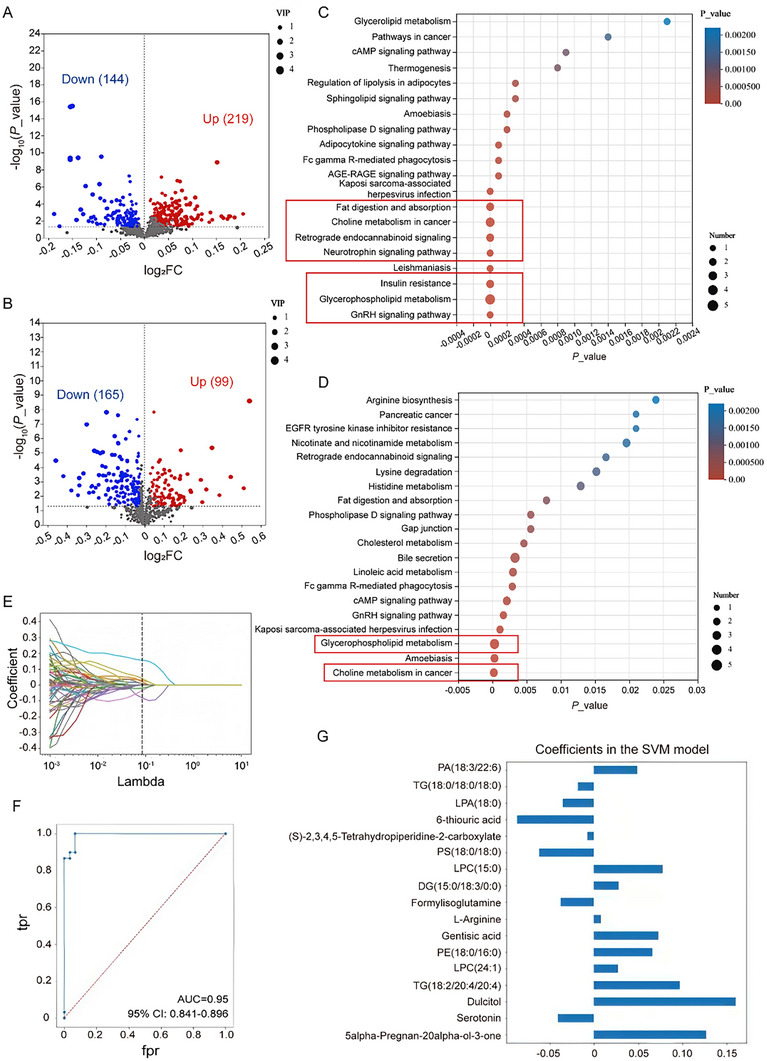
Dysregulated metabolites and candidate biomarker selection in preterm cerebral palsy. (A‐B) Volcano plots showing differential lipids (A) and polar metabolites (B) between preterm infants with CP (n = 30) and matched preterm controls (n = 30). Each dot represents one metabolite; the x‐axis indicates fold change (log_2_FC), the y‐axis represents significance (‐log_10_ *p* value), and dot size reflects the VIP score. Metabolites on the left are downregulated, while those on the right are upregulated. (C‐D) KEGG pathway enrichment bubble plots for differential lipids (C) and polar metabolites (D). The x‐axis shows enrichment significance (*p* value), the y‐axis lists KEGG pathways, bubble size represents the number of metabolites mapped, and bubble color corresponds to enrichment significance. (E) Lasso regression analysis identifying 17 key differential metabolites. (F) ROC curve of the SVM classification model constructed using selected metabolites, showing robust diagnostic performance (AUC = 0.95). (G) Feature importance ranking of differential metabolites in the SVM model.

Liquid Chromatography‐Mass Spectrometry (LC‐MS) identified 664 metabolites and 372 lipids, classified by the Human Metabolome Database (HMDB) (Figure S2A,B). Pathway enrichment analysis revealed significant involvement of multiple metabolic pathways (Figure S2C,D). Differential expression analysis demonstrated 219 lipids and 99 polar metabolites upregulated, whereas 144 lipids and 165 polar metabolites were downregulated in CP compared with controls (Figure 1A,B), with OPLS‐DA showing clear group separation (Figure S2E–H). Enrichment highlighted pathways relevant to brain development, including GnRH signaling, glycerophospholipid metabolism, neurotrophin signaling, and choline metabolism (Figure 1C,D).

**FIGURE 2:**
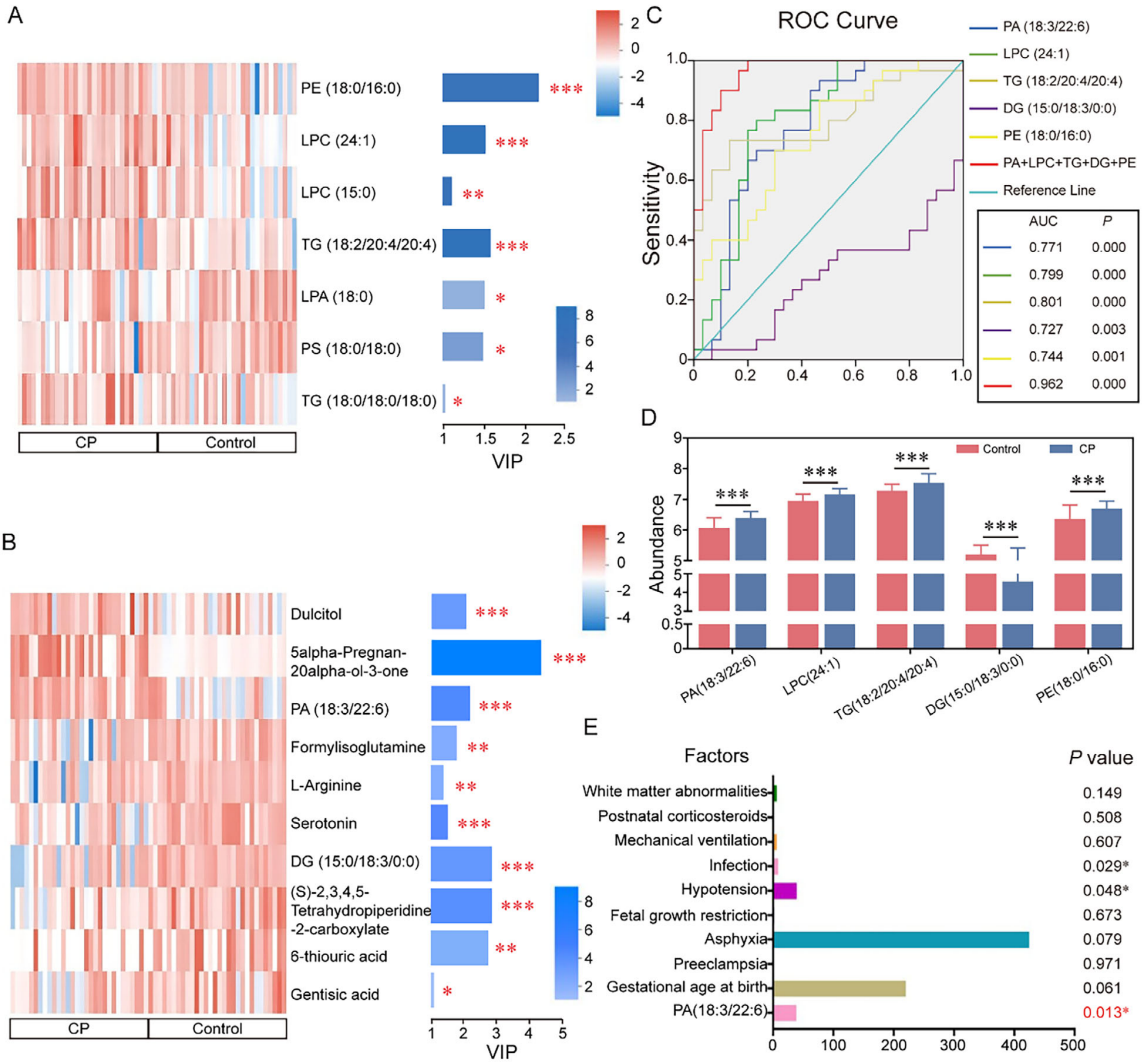
Identification and validation of candidate serum biomarkers for preterm cerebral palsy. (A‐B) Heatmaps (left) depicting relative expression of differential lipids (A) and polar metabolites (B) across individual samples, with corresponding VIP bar plots (right). Bar length reflects the contribution of each metabolite to group discrimination (VIP ≥ 1), and bar color indicates significance level (*p* value). (C) ROC curves of five candidate biomarkers, PA (18:3/22:6), LPC (24:1), TG (18:2/20:4/20:4), DG (15:0/18:3/0:0), and PE (18:0/16:0), demonstrating high predictive accuracy (AUC = 0.962). (D) Relative abundance of the five biomarkers between CP and control groups. (E) Binary logistic regression analysis confirming PA as an independent risk factor for preterm CP. *p < 0.05, **p < 0.01, ***p < 0.001.

LASSO regression selected 17 key metabolites that were incorporated into an SVM model, which demonstrated strong discriminatory performance between CP cases and controls (AUC = 0.95; Figure 1E–G). Heatmaps and VIP plots illustrated expression patterns (Figure 2A,B). Five metabolites, PA (18:3/22:6), LPC (24:1), Triacylglycerol (TG) (18:2/20:4/20:4), Diacylglycerol (DG) (15:0/18:3/0:0), and Phosphatidylethanolamine (PE) (18:0/16:0), exhibited the highest individual discriminatory capacity, with a combined ROC AUC of 0.962 (Figure 2C,D).

To explore clinical relevance, we stratified CP cases by motor type and functional severity. Clinical subtyping showed spastic quadriplegia (23.3%) and dyskinetic CP (26.7%) as the most frequent (Figure S3A). Gross Motor Function Classification System (GMFCS) identified 21 lipids and 24 polar metabolites associated with severe CP (GMFCS IV‐V) (Figure S3B,C). Notably, only PA, LPC, and TG species were mapped to Kyoto Encyclopedia of Genes and Genomes (KEGG) pathways, suggesting mechanistic relevance. ROC analysis for severe CP achieved an AUC of 0.9, with PA and several LPC/TG species showing strong discrimination (Figure S3D,E and Table S3). Logistic regression analysis revealed a significant association between elevated serum PA levels and CP status (Figure 2E). Importantly, PA was identified as a central intermediate linking glycerophospholipid metabolism and triglyceride synthesis, participating in multiple dysregulated pathways (Figure S3F).

### 3.2 Identification of Serum Biomarkers for Early Diagnosis of Preterm CP

To validate these findings, we conducted a prospective study enrolling 30 preterm infants diagnosed with CP and 30 matched controls without neurodevelopmental disorders. Compared with controls, infants with CP had significantly lower birth weight and a higher frequency of twin pregnancies. Classic perinatal risk factors, including maternal preeclampsia, perinatal asphyxia, postnatal hypotension, mechanical ventilation, and early MRI‐detected WMI, were enriched in the CP cohort (Table 2).

**TABLE 2:** Analysis of clinical characteristics of the prospective cohort.

Clinical characteristics	CP group	Control group	*p* value
Demographic			
Male, n (%)	13 (43.3%)	18 (60%)	0.196
Gestational age at birth, wk, mean (SE)	31.2 (0.60)	32.0 (0.44)	0.337
Birth weight, g, mean (SE)	1429 (107)	1831 (126)	0.018^*^
Current age, m, mean (SE)	17.2 (1.28)	18.7 (0.84)	0.557
Twins, n (%)	6 (20%)	1 (3.3%)	0.044^*^
Maternal			
Maternal age, y, mean (SE)	32.2 (0.63)	30.6 (1.12)	0.210
Placenta previa, n (%)	2 (6.7%)	1 (3.3%)	0.554
Preeclampsia, n (%)	12 (40%)	5 (16.7%)	0.045^*^
Primigravida, n (%)	15 (50%)	9 (30%)	0.114
Antenatal magnesium sulphate, n (%)	7 (23.3%)	5 (16.7%)	0.519
Other accompanying diseases, n (%)	11 (36.7%)	5 (16.7%)	0.080
Perinatal			
Placental abruption, n (%)	1 (3.3%)	0	0.313
Chorioamnionitis, n (%)	3 (10%)	0	0.076
Cesarean delivery, n (%)	23 (76.7%)	20 (66.7%)	0.390
1‐min Apgar score, mean (SE)	4.7 (0.38)	5.8 (0.31)	0.034^*^
5‐min Apgar score, mean (SE)	6.0 (0.33)	6.9 (0.27)	0.048^*^
Fetal growth restriction, n (%)	5 (16.7%)	2 (6.7%)	0.424
Kernicterus, n (%)	13 (43.3%)	11 (36.7%)	0.598
Postnatal			
Hypotension, n (%)	9 (30%)	2 (6.7%)	0.020^*^
Infection, n (%)	13 (43.3%)	8 (26.7%)	0.176
Mechanical ventilation ≥ 7d, n (%)	11 (36.7%)	4 (13.3%)	0.037^*^
Chronic lung disease, n (%)	5 (16.7%)	4 (13.3%)	0.718
Postnatal corticosteroids, n (%)	8 (26.7%)	4 (13.3%)	0.197
Imaging			
White matter abnormalities, n (%)	16 (53.3%)	6 (20%)	0.015^*^
Cerebrospinal fluid space abnormalities, n (%)	10 (33.3%)	5 (17%)	0.116
Cerebellar abnormalities, n (%)	2 (6.7%)	0	0.246

*Note*: The chi‐square test was employed to compare the counting data in groups. For comparison of measurement data between groups, Student's t test was used for normality, and the Mann–Whitney U test was used for non‐normality. The statistically significant p values are highlighted in red.

**FIGURE 3:**
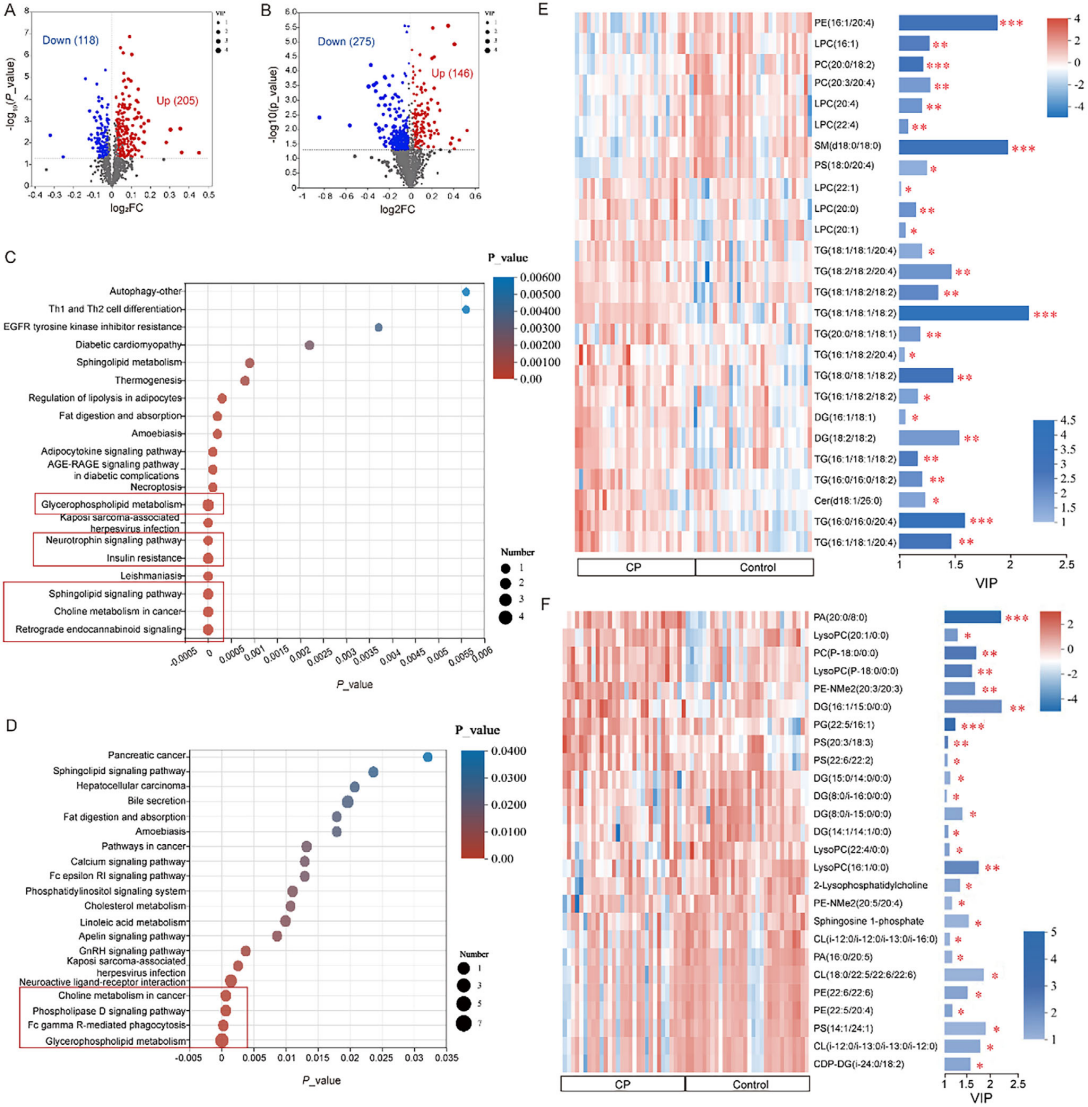
Prospective metabolomic and lipidomic profiling in preterm cerebral palsy. (A‐B) Volcano plots of differential lipids (A) and metabolites (B) between CP and matched controls. (C‐D) KEGG pathway enrichment bubble plots for lipids (C) and metabolites (D). The x‐axis denotes *p* value (enrichment significance), and bubble size represents the number of metabolites mapped to each pathway. (E‐F) Heatmaps and VIP bar plots of representative differential lipids (E) and metabolites (F) enriched in brain development‐related pathways. Heatmaps display relative abundance (rows: metabolites; columns: samples), while VIP bars indicate discriminatory contribution and statistical significance. *p < 0.05, **p < 0.01, ***p < 0.001.

Serum metabolomic and lipidomic profiling identified 1,971 metabolites and 400 lipids, annotated via HMDB (Figure S4A,B). KEGG analysis revealed lipid enrichment in glycerophospholipid, choline, and endocannabinoid, and metabolite enrichment in ABC transporters and amino acid metabolism (Figure S4C,D). OPLS‐DA revealed clear group separation, particularly in cationic mode (Figure S4E–H). Compared with controls, 205 lipids and 146 metabolites were upregulated, whereas 118 lipids and 275 metabolites were downregulated in CP (Figure 3A,B). KEGG enrichment linked differential metabolites to brain development‐related pathways, notably glycerophospholipid, sphingolipid, neurotrophin, and phospholipase D (Figure 3C,D). Heatmaps and VIP plots illustrated key differentially expressed molecules within these pathways (Figure 3E,F)

**FIGURE 4:**
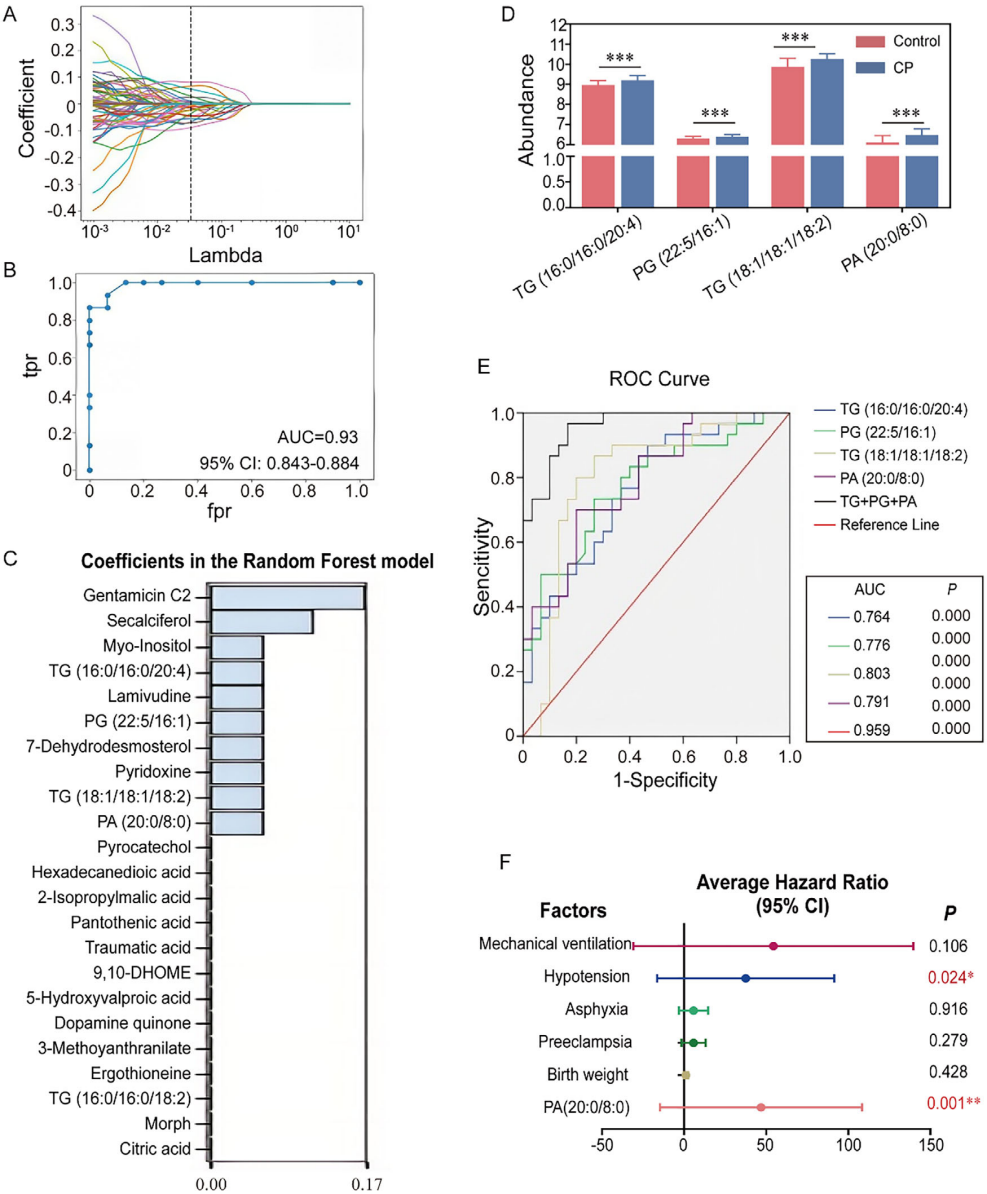
Identification and validation of candidate biomarkers in a prospective preterm cohort. (A) Lasso regression identifying 23 discriminative metabolites. (B‐C) Random forest classifier performance, including ROC curve analysis (B, AUC = 0.93) and feature importance ranking (C). (D‐E) Diagnostic evaluation of four candidate biomarkers, TG (16:0/16:0/20:4), PG (22:5/16:1), TG (18:1/18:2), and PA (20:0/18:0), showing relative abundance differences between groups (D) and ROC curve performance (E, AUC = 0.959). (F) Binary logistic regression analysis confirms elevated PA as an independent risk factor for CP. *p < 0.05, **p < 0.01, ***p < 0.001.

Lasso regression identified 23 discriminative metabolites, which were incorporated into a random forest model with strong discriminative performance (AUC = 0.93; Figure 4A–C). Four candidate metabolites, TG (16:0/16:0/20:4), Phosphatidylglycerols (PG) (22:5/16:1), TG (18:1/18:2), and PA (20:0/18:0), exhibited high discriminative ability between groups (AUC = 0.959; Figure 4D,E). Logistic regression analysis demonstrated a significant independent association between elevated PA levels and CP status (Figure 4F). Notably, PA elevation was detectable prior to clinical diagnosis, suggesting a potential early‐life association with subsequent CP development.

Finally, joint analysis of retrospective and prospective datasets consistently demonstrated enrichment of glycerophospholipid metabolism (Figure S5A,B), reinforcing its central involvement in CP‐associated metabolic dysregulation.

### 3.3 PA Elevation Impairs OPC Differentiation and Myelination

**FIGURE 5:**
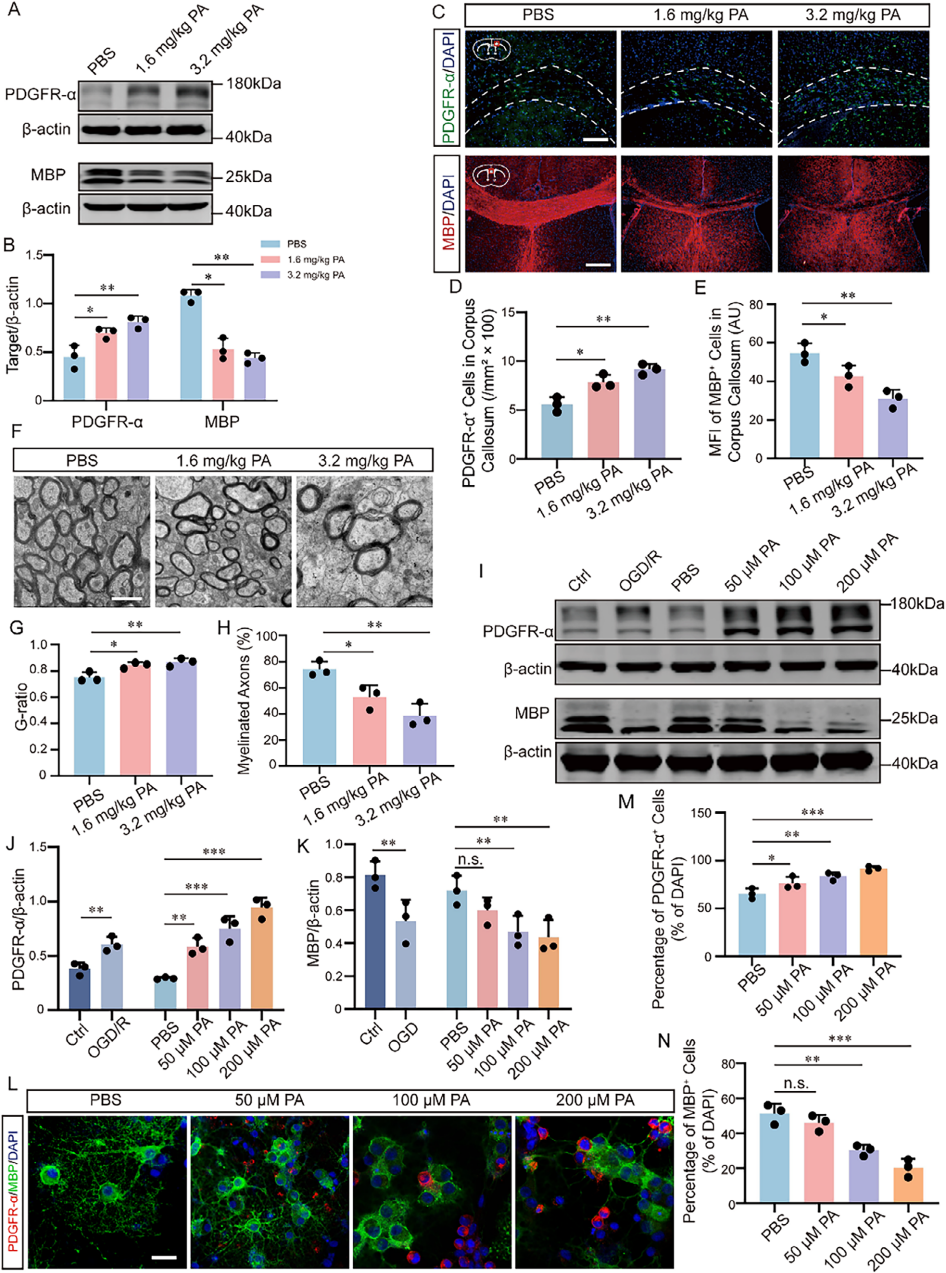
Elevated PA impairs OPC differentiation and myelination in vivo and in vitro. (A‐B) Western blot analysis of PDGFR‐α and MBP expression in the corpus callosum of mice intraperitoneally injected with PA for 7 consecutive days, with quantification (n = 3). (C‐E) Immunofluorescence staining showing increased PDGFR‐α^+^ cells and decreased MBP fluorescence intensity in the corpus callosum after PA injection, with quantification (n = 3; scale bar, 100 µm). The corpus callosum region is delineated by white dashed lines. PDGFR‐α was interpreted in the context of oligodendrocyte lineage localization and in conjunction with additional markers analyzed in parallel experiments. (F): TEM images of the myelin sheath of the corpus callosum of 3 groups of P14 mice, Bar = 1 µm. (G): G‐ratio statistical chart. (H): Percentage diagram of the number of myelinated axons. The measured number of axon myelin was n = 50/mouse. (I‐K) Western blot analysis of PDGFR‐α and MBP levels in primary OPCs following OGD/R (6 h OGD, 24 h reoxygenation) or PA treatment (50, 100, 200 µM; 72 h), with quantification (n = 3). (L‐N) Immunofluorescence analysis of PDGFR‐α^+^ and MBP^+^ cells in primary OPCs after PA treatment, Bar = 20 µm; n = 3). Data are presented as mean ± SD; *p < 0.05, **p < 0.01, ***p < 0.001.

We next investigated whether elevated PA contributes to WMI. Both brain tissue and serum from PWMI mice exhibited significantly higher PA levels than sham controls (Figure S6A). Elevated PA levels likely reflect a global metabolic response to HI stress rather than production by a single cell type. Given that oligodendrocyte lineage cells are primary targets of PWMI, we next examined how elevated PA impacts OPC biology. Similarly, OGD/R‐treated primary OPCs showed increased PA levels (Figure S6B). To determine functional consequences, mice were intraperitoneally injected with PA for 7 consecutive days (Figure S6C). Brain PA assays confirmed significant increases at 1.6 and 3.2 mg/kg (Figure S6D). PA‐treated mice exhibited elevated expression of the OPC marker platelet‐derived growth factor receptor alpha (PDGFR‐α) and reduced levels of the mature oligodendrocyte marker myelin basic protein (MBP), as assessed by WB and IF (Figure 5A–E). Ultrastructural analysis further revealed hypomyelination, characterized by increased g‐ratio values and reduced myelin density (Figure 5F–H).

We next validated these effects in vitro using primary OPC cultures (Figure S6E). PA treatment (50‐200 µM, 72 h) dose‐dependently increased PDGFR‐α and reduced MBP expression in primary OPCs (Figure 5I–K). IF further demonstrated reduced MBP^+^ and increased PDGFR‐α^+^ cells, with significant effects at 100 and 200 µM PA (Figure 5L–N).

To further validate the inhibitory role of PA, we reduced intracellular PA using enzyme inhibitors under OGD conditions (Figure S7A). Based on CCK‐8 assays, we established effective concentrations for each inhibitor: CI976 (Lysophosphatidic Acid Acyltransferase (LPAAT) inhibitor, 15 µM), R59949 (Diacylglycerol kinase (DGK) inhibitor, 25 µM), and FIPI (PLD inhibitor, 150 nM) (Figure S7B–D). Among the three biosynthetic pathways, inhibition of DGK (R59949) or PLD (FIPI) significantly decreased PDGFR‐α and increased MBP expression, whereas LPAAT inhibition (CI976) had no effect (Figure S7E–G). IF confirmed reduced PDGFR‐α^+^ and increased MBP^+^ cells after DGK or PLD inhibition (Figure S7H–J).

These results indicate that pathological PA accumulation, primarily mediated by DGK and PLD, disrupts OPC differentiation and myelination.

### 3.4 PA Interaction with TRIM59 Enhances its Protein Stability

**FIGURE 6:**
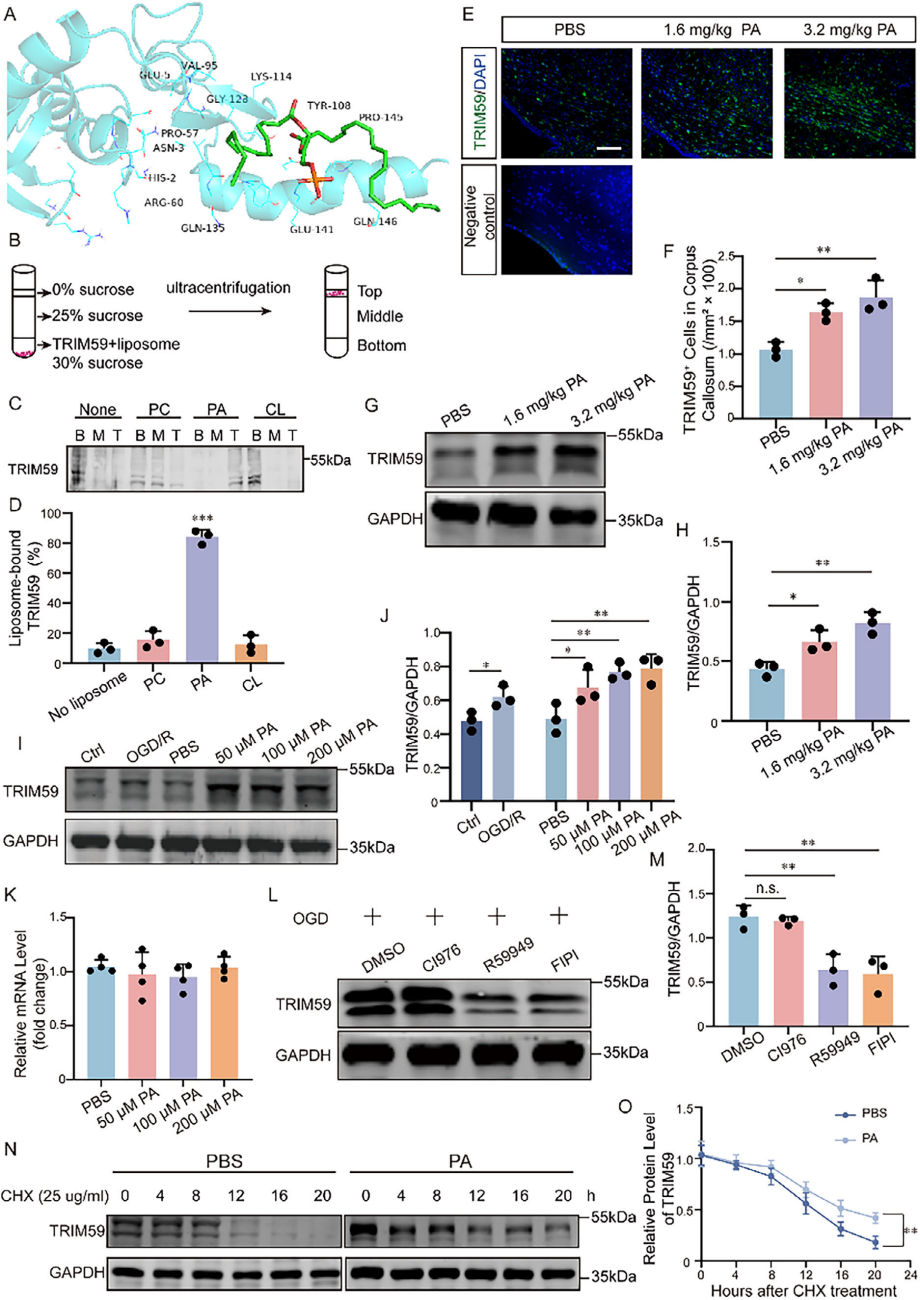
PA interaction with TRIM59 enhances its protein stability. (A) Molecular docking prediction of PA binding to TRIM59 at LYS114 using AutoDock. (B) Schematic of liposome flotation assay: purified TRIM59 was incubated with or without liposomes for 30 min, adjusted to 30% w/v sucrose, overlaid with two sucrose buffer layers, and subjected to ultracentrifugation. Top, middle, and bottom fractions were collected for Western blot analysis. (C‐D): Liposome flotation assay showing TRIM59 binding to PA‐enriched liposomes. Purified TRIM59 (1 µM) was incubated with 300 µM liposomes (100 nm in diameter) of different lipid compositions: PC only (100% PC), 20 mol% PA + 80 mol% PC, or 20 mol% CL + 80 mol% PC (n = 3). (E‐F) Immunofluorescence analysis of TRIM59^+^ cells in the corpus callosum of mice treated with PA (1.6 or 3.2 mg/kg, 7 days), with quantification. Insets show high‐magnification images from boxed regions. Scale bars: low magnification, 50 µm; high magnification, 20 µm. n = 3. Negative control images (secondary antibody only) were included to verify the specificity of TRIM59 immunofluorescence. (G‐H) Western blot analysis of TRIM59 protein levels in the corpus callosum of PA‐treated mice. n = 3. (I‐J) Western blot analysis of TRIM59 levels in OPCs following OGD/R or PA treatment (50‐200 µM), with quantification. n = 3. (K) qPCR analysis of TRIM59 mRNA levels in OPCs treated with PA. n = 4. (L‐M) Western blot analysis of TRIM59 protein levels in OPCs treated with PA synthesis inhibitors under OGD/R: DGK inhibitor R59949 (25 µM), PLD inhibitor FIPI (150 nM), or LPAAT inhibitor CI976 (15 µM). n = 3. (N‐O) Cycloheximide chase assays showing TRIM59 protein stability in OPCs treated with PA (100 µM). n = 3. Data are presented as mean ± SD; *p < 0.05, **p < 0.01, ***p < 0.001.

To identify downstream mediators of PA, we focused on TRIM59, an E3 ubiquitin ligase enriched in oligodendrocytes. Molecular docking predicted a PA‐binding site at LYS114 (Figure 6A). To validate this interaction, liposome flotation assays were performed using liposomes containing PA or control phospholipids. Cardiolipin (CL) was included to control for charge‐dependent interactions, and PC served as a nonspecific membrane control. Purified TRIM59 preferentially associated with PA‐containing liposomes, with minimal binding to CL‐ or PC‐containing liposomes (Figure 6B–D), indicating a specific interaction between PA and TRIM59.

We next examined whether PA regulates TRIM59 abundance in vivo and in vitro. In PWMI mice, TRIM59 protein levels were significantly increased in the ipsilateral corpus callosum (Figure S8A–D). Similarly, exogenous PA administration led to marked upregulation of TRIM59 in the corpus callosum (Figure 6E–H). Consistent results were observed in primary OPCs, where both OGD/R and PA treatment increased TRIM59 protein levels (Figure 6I–J), whereas qPCR revealed no change in mRNA (Figure 6K), suggesting post‐translational regulation. IF confirmed, increased TRIM59^+^ cells under OGD/R or PA exposure (Figure S8E,F). Importantly, inhibition of PA synthesis with the DGK inhibitor R59949 or the PLD inhibitor FIPI reduced TRIM59 protein expression, whereas the LPAAT inhibitor CI976 had no effect (Figure 6L–M). Cycloheximide chase assays further demonstrated that PA prolonged TRIM59 half‐life (Figure 6N,O). Collectively, these data demonstrate that PA directly interacts with TRIM59 and enhances its protein stability through post‐translational mechanisms.

### 3.5 PA‐TRIM59 Axis Promotes Olig2 Ubiquitination and Degradation

We next investigated whether TRIM59 targets differentiation‐associated transcription factors. Importantly, TRIM59 modulation selectively affected Olig2 protein levels without altering other major transcription factors involved in oligodendrocyte differentiation, including SOX10, Sip1, NKX2.2, Olig1, or Olig3 (Figure S9A,B), indicating a functionally selective substrate preference rather than a global effect on oligodendrocyte lineage regulators. Olig2 levels were markedly decreased in the PWMI corpus callosum and in OPCs exposed to OGD/R at all time points (3 h, 6 h, and 9 h OGD followed by 24 h reperfusion) (Figure S9C–F). Co‐IP confirmed increased Olig2 ubiquitination following OGD 6 h/R 24 h (Figure S9G).

**FIGURE 7:**
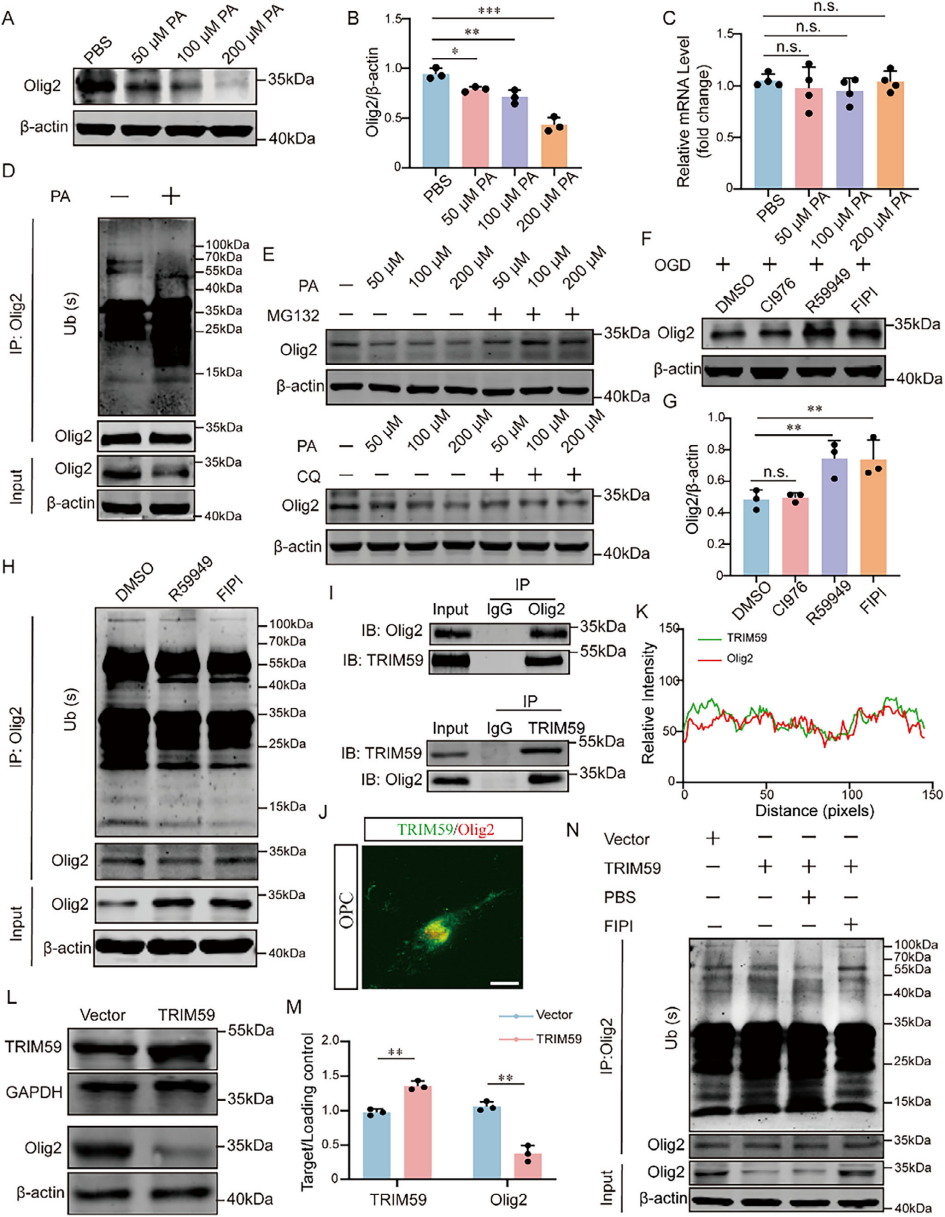
PA promotes TRIM59‐dependent ubiquitination and proteasomal degradation of Olig2. (A‐B) Western blot analysis and quantification of Olig2 protein levels in OPCs treated with increasing concentrations of PA (50 µM, 100 µM, 200 µM) (n = 3). (C) qPCR analysis showing no significant changes in Olig2 mRNA levels following PA treatment (n = 4). (D) Ubiquitination assay (IP/IB) demonstrating increased ubiquitination of Olig2 after 100 µM PA treatment. (E) Western blot analysis of Olig2 protein levels in OPCs treated with PA in the presence of MG132 (20 µM, 6 h; proteasome inhibitor) or chloroquine (CQ, 30 µM, 12 h; autophagy inhibitor), with quantification (n = 3). (F‐G) Western blot analysis and quantification showing that inhibition of PA synthesis with DGK inhibitor (R59949) or PLD inhibitor (FIPI), but not LPAAT inhibitor (CI976), restored Olig2 levels under OGD (n = 3). (H) Ubiquitination assay (IP/IB) showing reduced Olig2 ubiquitination after PA synthesis inhibition under OGD. (I) Co‐immunoprecipitation confirming physical interaction between TRIM59 and Olig2. (J‐K) Immunofluorescence images and colocalization analysis of TRIM59 and Olig2 in OPCs (scale bar = 5 µm). (L‐M) Western blot analysis and quantification showing decreased Olig2 protein upon TRIM59 overexpression in OPCs (n = 3). (N) Ubiquitination assay (IP/IB) demonstrating enhanced Olig2 ubiquitination following TRIM59 overexpression, which was reversed by PA synthesis inhibition (FIPI). Data are presented as mean ± SD. *p < 0.05, **p < 0.01, ***p < 0.001.

Exogenous PA reduced Olig2 protein but not mRNA (Figure 7A–C) and enhanced Olig2 ubiquitination (Figure 7D). Proteasome inhibition (MG132), but not autophagy inhibition (CQ), rescued Olig2 levels (Figure 7E). Inhibitors of PA synthesis (R59949, FIPI) restored Olig2 and reduced ubiquitination under OGD (Figure 7F–H). Mechanistically, TRIM59 physically interacts and colocalizes with Olig2 in OPCs (Figure 7I–K). TRIM59 overexpression in OPCs decreased Olig2 protein without altering mRNA levels (Figure 7L,M; Figure S10A), concomitant with increased Olig2 ubiquitination, which was reversed by PA synthesis inhibition with FIPI (Figure 7N). To directly test the requirement of TRIM59 in PA‐mediated Olig2 degradation, OPCs were treated with PBS, PA, or PA combined with siTRIM59. WB analysis showed that PA decreased Olig2 protein, whereas TRIM59 knockdown restored Olig2 to near‐baseline levels (Figure S10B,C). Linkage‐specific assays further revealed that TRIM59 predominantly mediates K48‐linked polyubiquitination of Olig2 (Figure S10D). Together, these results indicate that PA‐stabilized TRIM59 promotes K48‐linked ubiquitination and proteasomal degradation of Olig2.

### 3.6 Pharmacological Inhibition of PA Synthesis Restores Myelination and Rescues Behavioral Deficits in PWMI Mice

**FIGURE 8:**
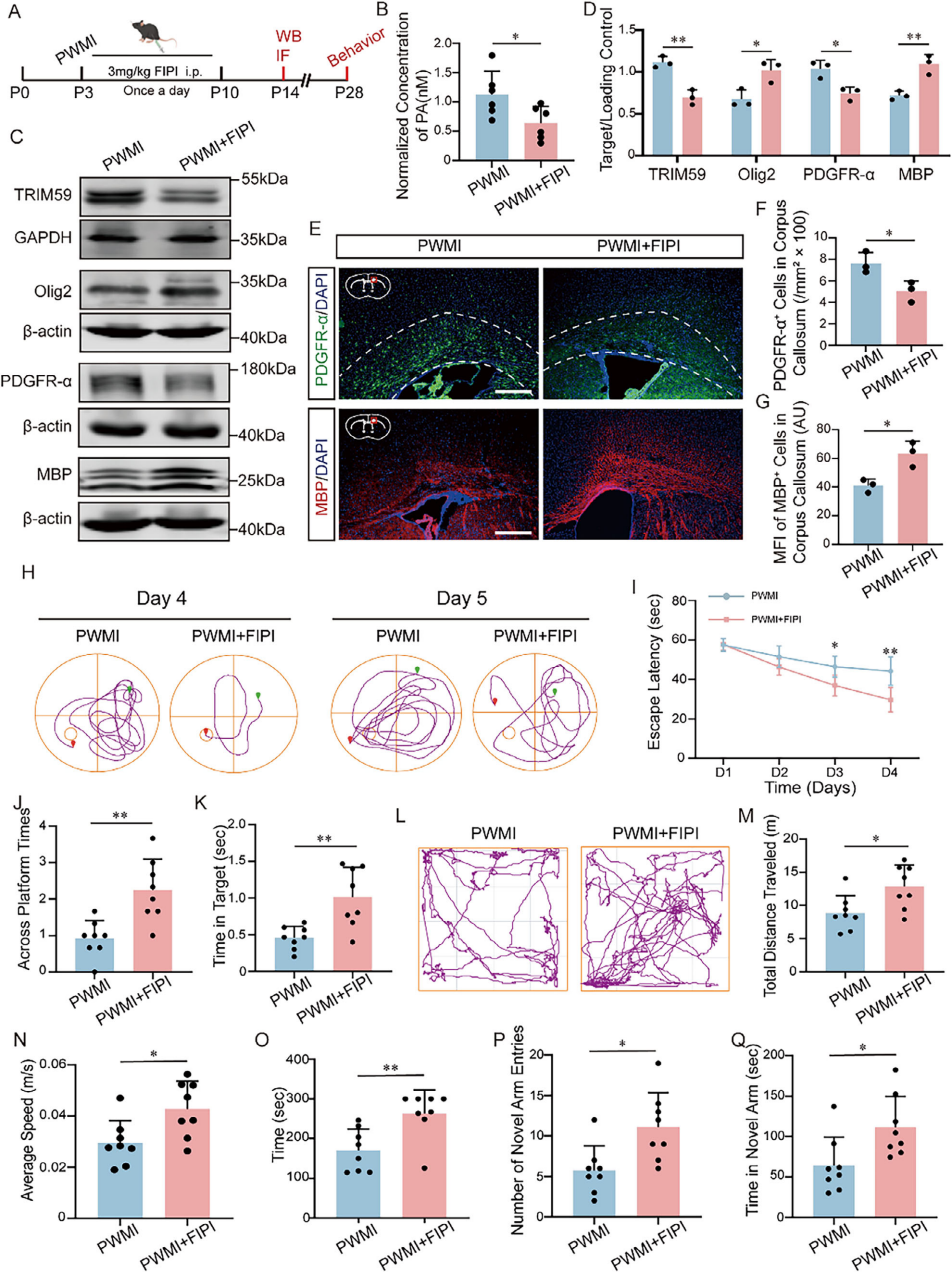
Pharmacological inhibition of PA synthesis restores myelination and rescues behavioral deficits in PWMI mice. (A) Schematic diagram of the experimental design. (B) Cerebral PA levels measured in PWMI and PWMI+FIPI groups (n = 3). (C‐D) Western blot analysis and quantification of TRIM59, Olig2, PDGFR‐α, and MBP protein levels in the corpus callosum of PWMI and PWMI+FIPI mice (n = 3). (E‐G) Immunofluorescence staining and quantification of PDGFR‐α^+^ cells and MBP fluorescence intensity in the corpus callosum of PWMI and PWMI+FIPI mice (scale bar, 100 µm; n = 3). The corpus callosum region is delineated by white dashed lines. (H) Representative swimming trajectories of mice in both groups during the Morris water maze (MWM) on days 4 and 5. (I) Escape latency during the 4‐day acquisition phase of the MWM, n = 8. (J‐K) Number of platform crossings and time spent in the target quadrant during the probe test on day 5 of the MWM, n = 8. (L) Representative locomotor trajectories in the open field test. (M‐N) Total distance traveled and average speed in the open field, n = 8. (O) Latency to fall in the rotarod test, n = 8. (P‐Q) Number of entries into the novel arm and time spent in the novel arm in the Y‐maze test, n = 8. Data are presented as mean ± SD. *p < 0.05, **p < 0.01.

To determine the in vivo relevance of the PA‐TRIM59‐Olig2 axis, we reduced cerebral PA levels in PWMI mice by intraperitoneal administration of the PA synthesis inhibitor FIPI (3 mg/kg daily, starting at P3 for 8 days; Figure 8A). A fluorometric assay confirmed that FIPI effectively lowered brain PA levels (Figure 8B). Western blot analyses further revealed that FIPI treatment significantly decreased TRIM59 expression and restored Olig2 levels in the corpus callosum compared with PWMI controls (Figure 8C,D). Consistently, suppression of PA synthesis reduced OPC accumulation, as reflected by decreased PDGFR‐α, and concomitantly enhanced myelination, as indicated by increased MBP expression (Figure 8C,D). IF validated these findings, showing fewer PDGFR‐α^+^ cells and markedly higher MBP fluorescence intensity in FIPI‐treated mice (Figure 8E–G). To evaluate the functional impact of PA suppression, we conducted behavioral assays. FIPI‐treated PWMI mice displayed improved locomotor activity (open field), enhanced motor coordination (rotarod), and significantly better spatial learning and memory performance (Morris water maze and Y‐maze) relative to untreated PWMI mice (Figure 8H–Q).

Together, these results demonstrate that pharmacological inhibition of PA synthesis mitigates TRIM59 upregulation and Olig2 degradation, thereby promoting OPC differentiation, restoring myelination, and rescuing neurobehavioral impairments in PWMI mice.

## 4 Discussion

In this study, we characterized the lipidomic and metabolomic alterations in preterm CP and identified several serum metabolites that robustly distinguish CP patients from matched preterm controls. Through integrated bioinformatics and machine learning analyses of retrospective and prospective clinical cohorts, we identified glycerophospholipid metabolism, particularly PA, as a critical metabolic alteration associated with CP, showing consistent elevation across independent cohorts and strong association with disease severity. These findings highlight the potential utility of PA as a candidate metabolic biomarker and support a pathogenic role of glycerophospholipid dysregulation in CP, consistent with targeted metabolomic analyses of postmortem brain tissue from patients with CP demonstrating marked disruption of glycerophospholipid metabolism [21]. Importantly, although PA elevation precedes clinical diagnosis in our prospective cohort and exerts direct effects on OPC differentiation in experimental models, PA should currently be viewed as a mechanistically relevant metabolic risk signal rather than a definitive causal determinant of CP. Although no significant sex differences were observed between groups in this study, sex‐specific metabolic regulation in PWMI and CP remains an important question that warrants investigation in larger, adequately powered cohorts.

Previous studies have shown that WMI is the predominant neuropathological substrate of preterm CP and is detectable by early neuroimaging [22, 23]. Our results extend this knowledge by providing biochemical evidence that altered phospholipid metabolism, particularly PA accumulation, may represent an upstream driver of OPC maturation failure. Phospholipids such as PE and LPC have already been implicated in myelin abnormalities [24, 25, 26], and we demonstrate that PA may play a central role as a signaling lipid regulating OPC differentiation and myelination.

Using a neonatal mouse model of PWMI, we validated that brain and serum PA levels are markedly elevated and coincide with impaired OPC differentiation and myelin formation. Both in vitro and in vivo, exogenous PA disrupted OPC maturation, whereas pharmacological inhibition of PA synthesis alleviated these defects. Although HI injury can affect multiple neural cell types, the concordant alterations in OPC differentiation and myelination observed in this study support a prominent contribution of white matter pathology to the behavioral phenotypes. Given PA's established role in peripheral nerve Schwann cell myelination and its regulation of mTORC1 signaling [18, 27, 28], our study provides the first evidence that PA accumulation is detrimental to CNS myelin development.

The upstream origin of elevated PA in CP likely reflects a systemic metabolic consequence of HI injury. Hypoxia is known to activate phospholipid remodeling enzymes, including PLD and DGK, leading to increased PA production across multiple cell types [29]. While circulating and brain PA may arise from diverse cellular sources, our data support a model in which elevated PA acts as a bioactive lipid signal within the injured brain microenvironment, with OPCs emerging as a particularly vulnerable cellular target rather than the primary source of PA elevation. In this broader neurobiological context, previous studies have implicated PA in diverse neural processes, including synaptic signaling, neuroinflammation, and membrane remodeling. However, its role in oligodendrocyte lineage regulation has remained poorly defined. Our findings extend this body of work by identifying a metabolically sensitive, ubiquitin‐dependent mechanism through which PA selectively destabilizes Olig2 under hypoxic stress, thereby directly linking lipid metabolic dysregulation to OPC differentiation failure in PWMI.

Mechanistically, we identified TRIM59, an E3 ubiquitin ligase, as a PA‐binding protein whose stability is enhanced by PA. Elevated TRIM59 in OPCs promoted Olig2 ubiquitination and degradation, thereby suppressing OPC differentiation. Although TRIM59 may interact with multiple proteins, our findings support a model in which Olig2 serves as a context‐dependent functional substrate in OPCs, owing to its central and rate‐limiting role in oligodendrocyte differentiation. Thus, TRIM59‐mediated destabilization of Olig2 is sufficient to account for the profound impairment in myelination observed in PWMI. Together, these data provide direct experimental support that PA enhances TRIM59 protein stability, which in turn promotes Olig2 ubiquitination and degradation, thereby impairing OPC differentiation. Olig2 is a master regulator of OPC lineage progression [30], and our findings place the PA‐TRIM59‐Olig2 axis as a novel regulatory pathway in developmental myelination. This observation resonates with prior reports of TRIM family proteins being upregulated in ischemia‐hypoxia models [31], supporting a role for stress‐induced TRIM59 in white matter pathology. Notably, this work reveals a previously unrecognized link between lipid metabolic dysregulation and TRIM59‐mediated ubiquitin signaling in WMI. Importantly, OPC differentiation and myelination are governed by integrated transcriptional and post‐translational regulatory networks; accordingly, the PA‐TRIM59‐Olig2 axis should be viewed as a metabolically responsive, injury‐associated pathway converging on Olig2 protein stability rather than a replacement for established developmental programs.

Although our lipid flotation and protein stability assays confirmed PA‐TRIM59 interaction, the exact binding site remains unknown. Based on analogies with other E3 ligases (e.g., Praja‐1) [32, 33], PA may compete with autoubiquitination sites to stabilize TRIM59, a hypothesis we will address in future mutational studies. While direct validation in human PWMI brain tissue is currently limited, the concordance between elevated serum PA levels in preterm CP infants and the mechanistic findings obtained from in vitro and in vivo experimental models supports the translational relevance of the PA‐TRIM59‐Olig2 pathway. In parallel with these mechanistic questions, the clinical translation of PA as a biomarker also warrants cautious interpretation. Although PA shows strong discriminatory performance in our cohort, the current sample size limits comprehensive multivariable adjustment for all perinatal confounders. Larger, prospectively collected cohorts will therefore be required to determine whether PA provides additive predictive value beyond established clinical risk factors.

Taken together, our findings identify PA as a key metabolic alteration associated with preterm CP and demonstrate its mechanistic role in PWMI through stabilization of TRIM59 and subsequent degradation of Olig2, thereby impairing OPC differentiation and myelin formation. The identification of the PA‐TRIM59‐Olig2 axis advances our understanding of PWMI pathogenesis and defines a biologically informed signaling pathway linking lipid metabolic dysregulation to oligodendrocyte lineage failure. From a translational perspective, these findings suggest that dysregulated PA signaling and its downstream effects on TRIM59‐Olig2 regulation represent a potential therapeutic vulnerability in PWMI. Rather than direct or global inhibition of TRIM59, strategies aimed at modulating PA metabolism or context‐specific TRIM59 activity within oligodendrocyte lineage cells may hold promise. Nevertheless, substantial future work will be required to rigorously assess feasibility, cell‐type specificity, developmental timing, and safety before clinical translation can be considered.

## Author Contributions

Conceptualization was performed by RQY, CR, and XYL. Data curation was carried out by YNL, XYL, MZ, and ZAL. Formal analysis was conducted by XYL, YNL, and MZ. Funding acquisition was secured by RQY, CR.Investigation was performed by XYL, YNL, MZ, YWL, MTL, and KSZ. The methodology was developed by YNL, XYL, MZ, RQY, ZAL, YWL, MTL, and KSZ. Project administration was managed by RQY, CR, and ZAL. Resources were provided by RQY. Supervision was undertaken by RQY, CR, and ZAL. Validation was performed by RQY, CR, and ZAL. Visualization was completed by MZ, XYL, and YNL. The original draft was written by XYL, YNL, and MZ, and the manuscript was reviewed and edited by RQY, XYL, YNL, and MZ. All authors approved the final version of the manuscript.

## Conflicts of Interest

The authors declare no conflicts of interest.

## Supporting information




**Supporting File 1**: advs74495‐sup‐0001‐SuppMat1.docx.

**Supporting File 2**: advs74495‐sup‐0002‐SuppMat2.docx.

**Supporting File 3**: advs74495‐sup‐0002‐Data.zip.

## Data Availability

The data that support the findings of this study are available from the corresponding author upon reasonable request.;
