# Supplementary material for: Phosphatidic Acid‐TRIM59‐Olig2 Signaling Couples Metabolic Dysfunction to Myelination Failure in PWMI
Source: Adv Sci (Weinh). 2026 Feb 18;13(25):e21296. doi: 10.1002/advs.202521296 (PMC13137782; doi:10.1002/advs.202521296)
Supplement: Supplementary file 1 — Supporting File 1: advs74495‐sup‐0001‐SuppMat1.docx. [file ADVS-13-e21296-s001.docx]

**Supplementary Methods**

**1. Retrospective Case-Control Study**

**1.1 Study population**

Thirty children aged 1-4 years with CP receiving rehabilitation at the Pediatric Rehabilitation Department of the Affiliated Hospital of Xuzhou Medical University were enrolled. Thirty age-matched preterm infants without neurodevelopmental disorders served as controls.

**1.2 Inclusion criteria for CP group**

1) Age 1-4 years;

2) Preterm birth (28 ≤ gestational weeks < 37, 1000 g ≤ birth weight ≤ 2499 g);

3) Clinical diagnosis of CP confirmed by experienced pediatricians according to the 2006 international CP criteria and 2014 Chinese CP guidelines;

4) Written informed consent obtained from legal guardians.

**1.3 Exclusion criteria for CP group**

1) Known genetic or metabolic disorders mimicking CP;

2) Other neurological disorders causing motor or cognitive dysfunction (e.g., autism, epileptic encephalopathy);

3) Severe systemic illness, such as liver/kidney dysfunction, acute infection, or trauma.

**1.4 Inclusion/exclusion criteria for controls**

Controls were age-matched preterm infants meeting the same gestational age and birth weight criteria without neurodevelopmental disorders and with written informed consent. Exclusion criteria were severe systemic illness.

**1.5 Clinical data collection**

Demographic data (age, sex, birth weight), maternal information (maternal age, obstetric history), perinatal characteristics (intrauterine growth restriction, birth asphyxia, maternal preeclampsia, placenta previa), and neonatal interventions (mechanical ventilation, corticosteroids) were recorded. CP severity was assessed using the Gross Motor Function Classification System (GMFCS).

**1.6 Blood collection and serum preparation**

Peripheral venous blood (2-3 mL) was collected between 08:00 and 09:00 into serum separator tubes. Samples were allowed to clot at room temperature for 2 h, centrifuged at 3000 rpm for 15 min at 4 °C, and serum was aliquoted into 0.5 mL tubes and stored at -80 °C until analysis.

**2. Prospective Clinical Cohort Study**

**2.1 Study population**

Preterm infants admitted to the NICU at Xuzhou Medical University within 72 hours after birth were enrolled. Infants later diagnosed with CP formed the patient group, while neurologically normal preterm infants served as controls. Each group included 30 infants.

**2.2 Inclusion criteria**

1) Preterm birth (28 ≤ gestational weeks < 37, 1000 g ≤ birth weight ≤ 2499 g);

2) Admission to the NICU at Xuzhou Medical University;

3) Written informed consent obtained from legal guardians.

**2.3 Exclusion criteria**

1) Prenatal diagnosis of congenital malformations or genetic defects;

2) Severe systemic illness or other neurological disorders.

**2.4 Clinical data collection**

Demographic, maternal, and perinatal data were recorded, including intrauterine growth restriction, birth asphyxia, maternal preeclampsia, placenta previa, placental abruption, or chorioamnionitis. Postnatal data included hypotension, infection, mechanical ventilation, and corticosteroid therapy. Neuroimaging data within the first 3 months were collected.

**2.5 Follow-up**

Infants were regularly followed by experienced pediatricians until CP diagnosis was confirmed or neurodevelopmental disorders were excluded. At each follow-up, neuroimaging, neurological assessments, and neurodevelopmental scoring were recorded.

**2.6 Blood collection and serum preparation**

Peripheral venous blood (1-1.5 mL) was collected into serum separator tubes, allowed to clot at room temperature for 2 h, centrifuged at 3000 rpm for 15 min at 4 °C, and serum was aliquoted into 0.5 mL tubes and stored at -80 °C until analysis to prevent repeated freeze-thaw cycles.

**3. Lipidomic Analysis**

**3.1 Sample preparation**
For lipid analysis, 200 µL serum was extracted with 80 µL methanol and 400 µL MTBE. After vortexing (30 s), sonication (40 Hz, 30 min, 5 °C), precipitation (-20 °C, 30 min), and centrifugation (13,000g, 15 min, 4 °C), 350 µL of supernatant was dried under nitrogen, reconstituted in 100 µL 2-propanol:acetonitrile (1:1), vortexed, sonicated (5 min, 5 °C), centrifuged (13,000g, 10 min, 4 °C), and injected into LC-MS/MS. QC samples were prepared as above.

**3.2 LC-MS/MS acquisition**
Lipid separation was performed on a Thermo UHPLC Vanquish Horizon system with an ACQUITY BEH C18 column. MS data were acquired using Thermo Q Exactive MS in both ion modes. Source conditions: heater 370 °C; sheath gas 60 psi; aux gas 20 psi; ISVF -3000 V (negative)/+3000 V (positive); collision energy 20-40-60 eV. DDA mode was applied, with scan range 200-2000 m/z.

**3.3 Data processing and annotation**
Data were analyzed in LipidSearch (Thermo). Precursor and fragment mass tolerances were set to 5 ppm. Features detected in ≥80% of samples were retained. Minimum values were imputed, normalized by sum, and features with QC RSD >30% were removed. Identification was based on MS/MS fragments, using grades A-D for ID quality filter.

**4. Metabolomic Analysis**

**4.1 Sample preparation**
For metabolite analysis, 50 µL serum was extracted with 400 µL methanol: acetonitrile (1:1, v/v) containing 0.02 mg/mL 2-Chloro-L-Phenylalanine. Extracts were vortexed (30 s), sonicated (40 Hz, 30 min, 5 °C), precipitated (-20 °C, 30 min), and centrifuged (13,000g, 15 min, 4 °C). Supernatants were dried under nitrogen, reconstituted in 120 µL acetonitrile: water (1:1), vortexed, sonicated (5 min, 5 °C), centrifuged (13,000g, 10 min, 4 °C), and injected into LC-MS/MS. QC samples were prepared by pooling 20 μL aliquots from each serum sample.

**4.2 LC-MS/MS acquisition**
Chromatographic separation was performed on a Thermo UHPLC system with an ACQUITY BEH C18 column (100 mm × 2.1 mm, 1.7 µm). Data were acquired using a Thermo Q Exactive MS with an ESI source in positive and negative ion modes. Source conditions: heater 400 °C; sheath gas 40 psi; aux gas 30 psi; ISVF -2800 V (negative)/3500 V (positive); collision energy 20-40-60 eV. Data-dependent acquisition (DDA) mode was applied with a scan range of 70-1050 m/z.

**4.3 Data processing and annotation**
Raw data were processed using Progenesis QI (Nonlinear Dynamics, Waters). Features detected in ≥80% of samples were retained. Missing values were imputed, features normalized by sum, and those with QC RSD >30% removed. Annotation was based on accurate mass, MS/MS spectra, and isotope ratio, using HMDB (<http://www.hmdb.ca/>) and Metlin (<https://metlin.scripps.edu/>), with a mass tolerance of ±10 ppm. Metabolites with MS/MS fragment score >30 were considered confidently identified.

**5. Statistical and Pathway Analysis**

Multivariate analysis was conducted using the *ropls* R package on Majorbio Cloud (<https://cloud.majorbio.com>). PCA was applied for unsupervised clustering, and OPLS-DA for supervised group discrimination. Model validity was assessed by R^2^ and Q^2^ values. Variable importance in projection (VIP) scores were calculated. Differential metabolites/lipids were defined as VIP >1 and *p* <0.05 (Student’s *t*-test). Enrichment and pathway analyses were performed using KEGG (<http://www.genome.jp/kegg/>) with Fisher’s exact test (*scipy.stats* package, Python). Diagnostic performance was assessed by SVM classification and ROC curve analysis (SPSS v22.2).

**6. Isolation and culture of primary OPCs**
Neonatal mice (P0-P2) were sacrificed by hypothermia anesthesia. After surface sterilization, cerebral cortices were dissected in ice-cold DMEM/F12 (Gibco, 11965092, USA). Meninges and non-cortical tissue were carefully removed. Cortical tissue was minced and mechanically dissociated into single-cell suspensions, which were filtered through a 40 µm cell strainer. Cells were pelleted by centrifugation (1200 rpm, 5 min), resuspended, and plated onto poly-D-lysine (Sigma-Aldrich, P6407, USA)-coated T25 flasks at a density of 1.0-2.0 × 10^6 cells/flask. Mixed glial cultures were maintained in DMEM/F12 supplemented with 10% fetal bovine serum in a humidified incubator (5% CO_2_, 37 °C), with medium replaced every 2 days.

After 7-9 days, OPCs were separated from the mixed glia by orbital shaking. Cells were detached using enzyme-free dissociation buffer, collected by centrifugation, and replated at 2 × 10^4^ cells/mL. For proliferation, cultures were maintained in medium supplemented with basic fibroblast growth factor (bFGF, 10 ng/mL). For differentiation, cells were switched to serum-free medium containing 15 nM triiodothyronine (T3, Sigma-Aldrich, P6397, USA), without PDGF or bFGF, and cultured for 5-10 days with medium changes every 3 days.

**7. Immunofluorescence staining**
Coronal cryosections of mouse brains (20 µm) were prepared and treated with 0.3% Triton X-100 and 5% bovine serum albumin (BSA, VICMED, VIC018, Australia) in PBS for 30 min at 37 °C to block nonspecific binding and permeabilize membranes. Cultured cells were fixed with 4% paraformaldehyde (Solarbio, P1110, China) for 20 min at room temperature, rinsed three times with PBS, and subsequently subjected to the same blocking and permeabilization procedure. Samples were incubated overnight at 4 °C with the following primary antibodies: MBP (Santa Cruz, sc-376995; 1:200), PDGFR-α (Abcam, ab203491; 1:300), Olig2 (Millipore, MABN50; 1:500), and TRIM59 (Proteintech, 28575-1-AP; 1:200). After three PBS washes, Alexa Fluor-conjugated secondary antibodies (488 or 594, Abcam; 1:500) were applied for 1 h at room temperature in the dark. Nuclei were counterstained with DAPI (Solarbio, C0060, China) for 5 min.

Fluorescence images were captured using an Olympus BX53 microscope with a DP80 camera. Exposure parameters were kept constant across groups to ensure comparability. At least three independent biological replicates were analyzed for each condition.

**8. Western blot analysis**
Brain tissue samples and cultured cells were harvested and lysed in ice-cold RIPA buffer (Beyotime, P0013B, China) containing 1 mM PMSF (Beyotime, ST506, China). Tissue samples were additionally homogenized by ultrasonication. Cytoplasmic protein fractions were obtained using a cytoplasmic protein extraction kit (Beyotime, P0027, China) according to the manufacturer’s instructions. Protein concentrations were quantified with a BCA assay kit (Beyotime, P0012, China). Equal amounts of protein (20-30 µg) were mixed with 5× SDS sample buffer, denatured at 95 °C for 5 min, and separated on 10% SDS-PAGE gels. Proteins were transferred onto 0.22 µm PVDF membranes (Millipore, IPVH00010, USA) by wet transfer and blocked in PBST containing 5% non-fat milk for 1 h at room temperature.

The membranes were incubated overnight at 4 °C with primary antibodies, including anti-MBP (Santa Cruz, sc-376995, 1:1000), anti-PDGFR-α (Abcam, ab203491, 1:1000), anti-TRIM59 (Proteintech, 28575-1-AP, 1:1000), anti-Olig2 (Millipore, MABN50; 1:1000), anti-Ubiquitin (Santa Cruz, sc-8017, 1:200), anti-Olig1 (Santa cruz, sc-373679, 1:100), anti-Olig3 (Abmart, T510208, 1:500), anti-SOX10 (Santa cruz, sc-365692, 1:100), anti-Sip1 (Santa cruz, sc-271984, 1:100), anti-NKX2.2 (Santa cruz, sc-398951, 1:100), β-actin (Proteintech, 66009-1-Ig, 1:5000) and GAPDH (Abmart, M20006, 1:5000). Negative control blots were incubated with secondary antibodies alone to assess nonspecific binding.

After washing, membranes were incubated for 1-2 h at room temperature with infrared dye-conjugated secondary antibodies (IRDye® 680RD goat anti-rabbit IgG, 1:10,000; IRDye® 800CW goat anti-mouse IgG, 1:20,000; LI-COR). Protein bands were visualized using an Odyssey infrared imaging system (LI-COR Biosciences, USA). Signal intensities were analyzed with ImageJ software (NIH, version 1.8.0). Each experiment was performed in at least three independent replicates.

**9. siRNA transfection**

Primary oligodendroglial cells (~60-70% confluency) were transfected with siTRIM59 using SilentFect™ Lipid Reagent (Bio-Rad, 1703361) in serum-free DMEM/F-12. Briefly, siRNA and lipid reagent were separately diluted, combined to form complexes, and added to cells for 6-8 h. The medium was then replaced with complete growth medium. Cells were harvested 24-96 h post-transfection for RNA or protein analysis.

**10. TRIM59 protein purification and liposome flotation**

**Protein expression and purification:** Trim59 coding sequence was codon-optimized, synthesized, and cloned into pGS21T with N-terminal His-GST tag. The construct was transformed into Rosetta (DE3) cells, induced with 0.5 mM IPTG at OD_600_ ~0.6, and expressed at 15 °C for 15 h. Cells were lysed in Buffer D (20 mM Tris-HCl, 50 mM NaCl, 0.1% Triton X-100), centrifuged, and the supernatant was applied to a GST affinity column. Insoluble protein in inclusion bodies was solubilized with 8 M urea and refolded via stepwise dialysis in refolding buffer (50 mM Tris, 50 mM NaCl, 500 mM L-arginine, 1 mM GSH, 3 mM GSSG, pH 8.0). Protein concentration was determined by BCA assay (~0.9 mg/mL). **Liposome preparation:** PC, PA, and CL lipids were mixed, dried, and rehydrated in 20 mM HEPES pH 7.5, 150 mM KCl. Liposomes were subjected to freeze-thaw cycles and extruded through polycarbonate membranes (50-1000 nm) using a MiniExtruder (Avanti). **Flotation assay:** TRIM59 (1 µM) was incubated with liposomes (300 µM) at 37 °C for 20 min. Samples were adjusted to 30% sucrose (bottom fraction), overlaid with 25% sucrose (middle) and buffer (top), and centrifuged at 240,000 × g for 2 h at 4 °C. Fractions were collected and analyzed by western blot with anti-TRIM59 antibody. The proportion of TRIM59 in the top fraction was quantified to evaluate lipid binding.

**11. Behavioral Assessments**
**11.1 Morris water maze (MWM):** Spatial learning and memory were evaluated using the MWM paradigm as previously described with modifications. The apparatus consisted of a circular pool (120 cm diameter, 40 cm height) filled with opaque water at 25 °C. A hidden escape platform (8 cm diameter) was placed 0.5–1 cm below the water surface in a fixed quadrant, with distal visual cues positioned around the testing area. Mice (n = 8 per group) were habituated to the testing environment for 2–3 h prior to trials conducted between 9:00 and 12:00. Training comprised four trials per day (two sessions, two trials/session) for 4 consecutive days, during which mice were released from random start positions facing the wall. If the platform was not located within 60 s, mice were guided to it and allowed to remain for 30 s. On day 5, a probe trial was performed in the absence of the platform. Swimming trajectories were monitored by a video tracking system (ANY-maze, Stoelting, USA), and escape latency, platform crossings, and quadrant occupancy were quantified (Ethovision 3.1, Noldus).

**11.2 Open field test:** Locomotor activity and exploratory behavior were assessed in a square arena (50 × 50 × 30 cm). Each animal was placed in the center and allowed to explore freely for 5 min while movement was recorded by ANY-maze. Parameters including total distance, mean velocity, and time spent in central versus peripheral areas were analyzed. The apparatus was cleaned with 75% ethanol between trials.

**11.3 Y-maze:** The Y-maze consisted of three arms of equal dimensions. In the training phase, one arm was blocked and mice explored the other two arms for 3 min. After 4 h, a test session was conducted with all arms open, allowing free exploration for 3 min. Spontaneous alternation, novel arm entries, and exploration time were measured.

**11.4 Rotarod test:** Motor coordination and balance were examined using an accelerating rotarod. Mice were pretrained with gradually increasing rotation speeds (4 rpm for 1 min, 10 rpm for 2 min, and 30 rpm for 3 min) separated by 15-min rest intervals. Twenty-four hours later, the test session began with the rod accelerating to 20 rpm. The latency to fall was recorded, with a maximum cut-off of 300 s. Each mouse completed three trials, and the mean latency was used for analysis.
