# Supplementary material for: Phosphatidic Acid‐TRIM59‐Olig2 Signaling Couples Metabolic Dysfunction to Myelination Failure in PWMI
Source: Adv Sci (Weinh). 2026 Feb 18;13(25):e21296. doi: 10.1002/advs.202521296 (PMC13137782; doi:10.1002/advs.202521296)
Supplement: Supplementary file 2 — Supporting File 2: advs74495‐sup‐0002‐SuppMat2.docx. [file ADVS-13-e21296-s003.docx]

**Supplementary Figure 1**


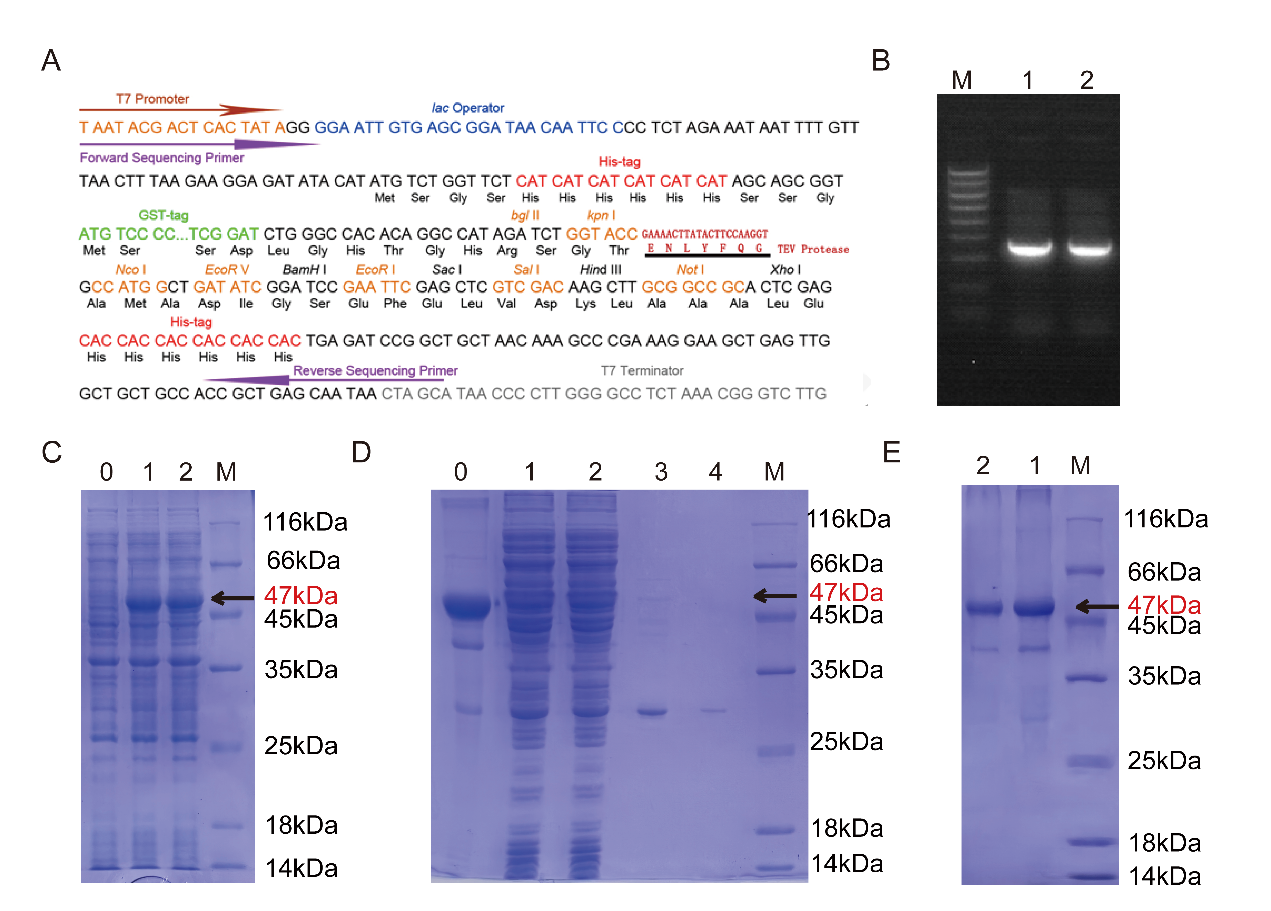


**Figure S1. Validation of TRIM59 protein purification.** (A) Schematic diagram of the TRIM59 expression vector. (B) PCR amplification of TRIM59. (C) Expression of TRIM59-pGS21T in E. coli: 0, non-induced culture; 1, induced colony #1; 2, induced colony #2. (D) GST affinity purification of TRIM59: 0, pellet after sonication and centrifugation of induced culture; 1, supernatant after sonication and centrifugation; 2, flow-through from GST purification column; 3, wash fraction with buffer D; 4, eluted target protein. (E) Refolding of TRIM59 protein: 1, supernatant after solubilization of inclusion bodies with 8 M urea followed by sonication and centrifugation; 2, supernatant after refolding and concentration.

**Supplementary Figure 2**


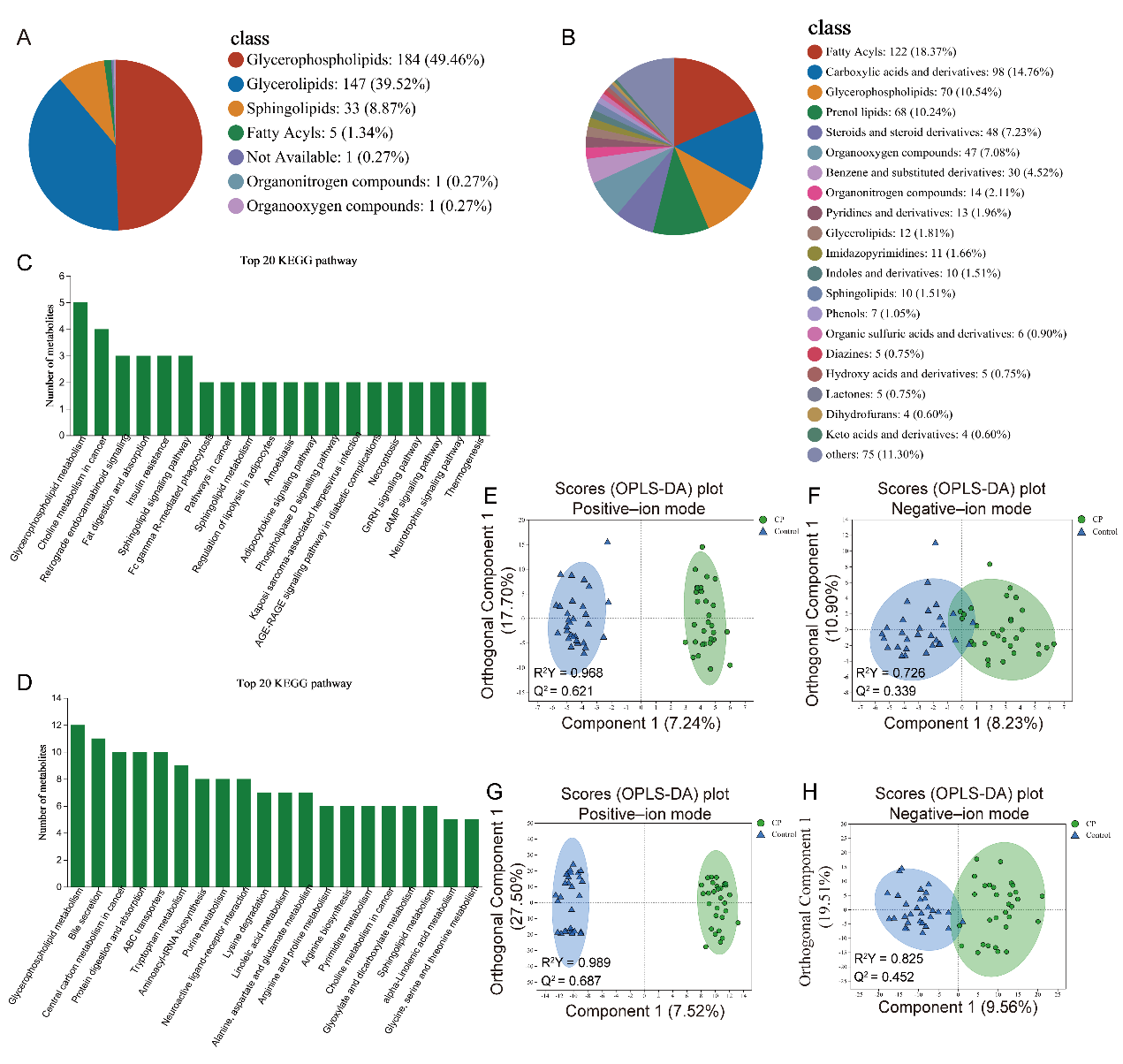


**Figure S2. Classification of identified compounds, pathway activity ranking, and OPLS-DA model validation.** (A-B) HMDB-based classification of identified lipids (A) and polar metabolites (B). Each pie slice denotes an HMDB class and slice area indicates the relative proportion of identified compounds assigned to that class. (C-D) Top 20 KEGG metabolic pathways ranked by activity for the lipidome (C) and metabolome (D). Bars are ordered from left to right by the number of identified metabolites mapped to each pathway (high → low); bar height reflects the number of mapped metabolites, with taller bars indicating greater pathway involvement among identified compounds. (E-H) OPLS-DA score plots for lipidomics (E-F, positive and negative ion modes) and metabolomics (G-H, positive and negative ion modes). OPLS-DA applies orthogonal rotation to remove variation unrelated to group separation, thereby enhancing discrimination between groups. “Comp1” denotes the first predictive component and “Orthogonal Comp1” denotes the first orthogonal component. R^2^Y and Q^2^ report model fit and predictive ability, respectively; values closer to 1 indicate a more stable and predictive model.

**Supplementary Figure 3**


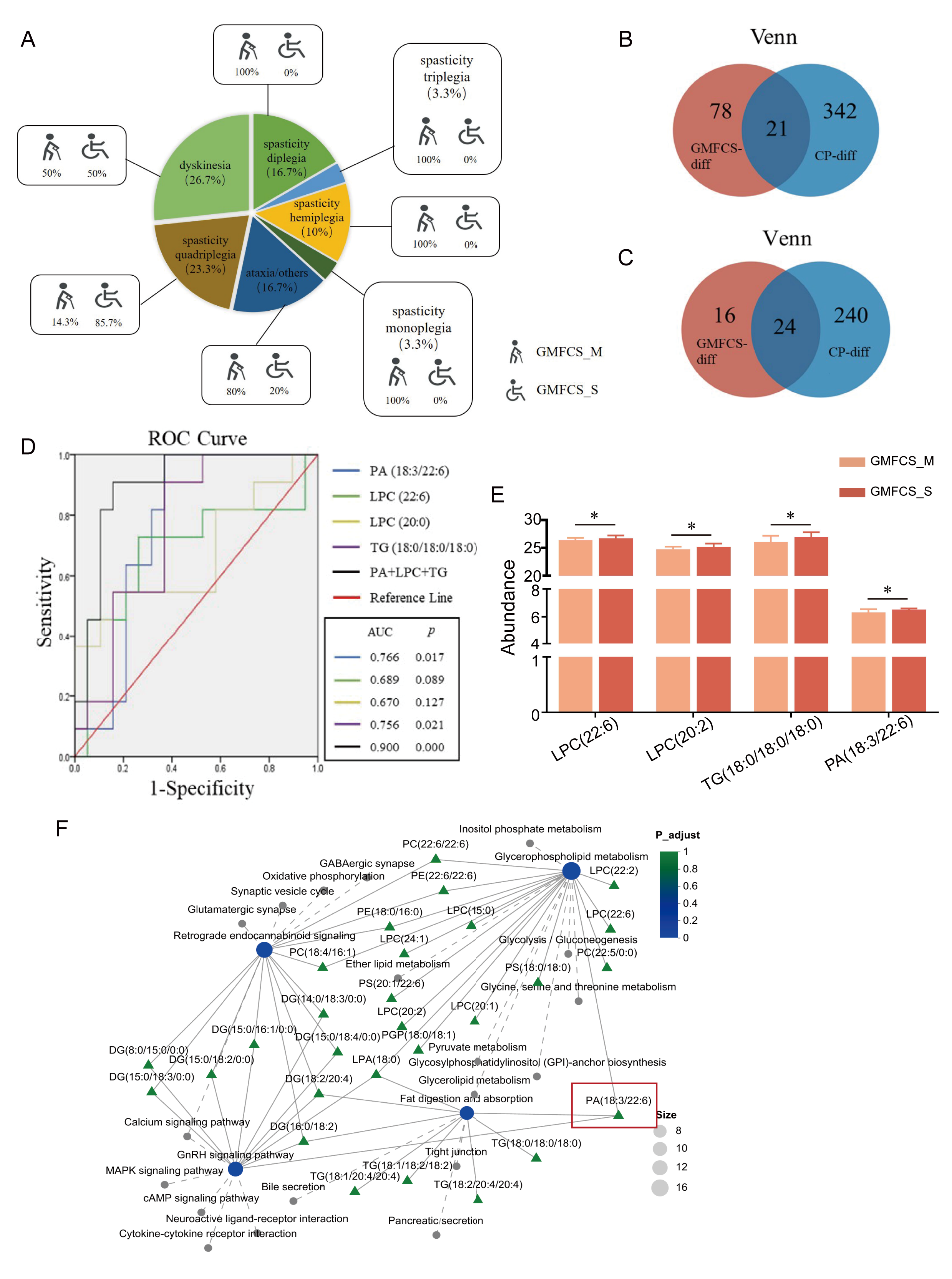


**Figure S3. Clinical subtyping, overlap of differential metabolites, and biomarker performance for severe CP.** (A) Clinical stratification by GMFCS: GMFCS IV-V were defined as severe CP (GMFCS_S) and GMFCS I-III as non-severe CP (GMFCS_M). (B-C) Venn diagrams for lipidomics (B) and metabolomics (C) showing overlaps among differential sets. “GMFCS_diff” denotes metabolites differentially abundant between severe and non-severe CP, and “CP_diff” denotes metabolites differentially abundant between CP cases and controls. Overlapping areas indicate metabolites shared between comparisons. (D) ROC curve analysis for four candidate biomarkers discriminating severe (GMFCS_S) from non-severe (GMFCS_M) cases, achieving an overall diagnostic performance of AUC = 0.90. (E) Relative abundance plots of the four candidate biomarkers between GMFCS_S and GMFCS_M groups. (F) KEGG functional pathway network. Triangular (green) nodes represent individual metabolites; circular nodes represent KEGG pathways. Node color corresponds to enrichment significance (smaller *P* values shown in a darker/distinct color), and node size indicates the number of metabolites mapped to that pathway. Edges indicate mapping relationships between metabolites and pathways.

**Supplementary Figure 4**


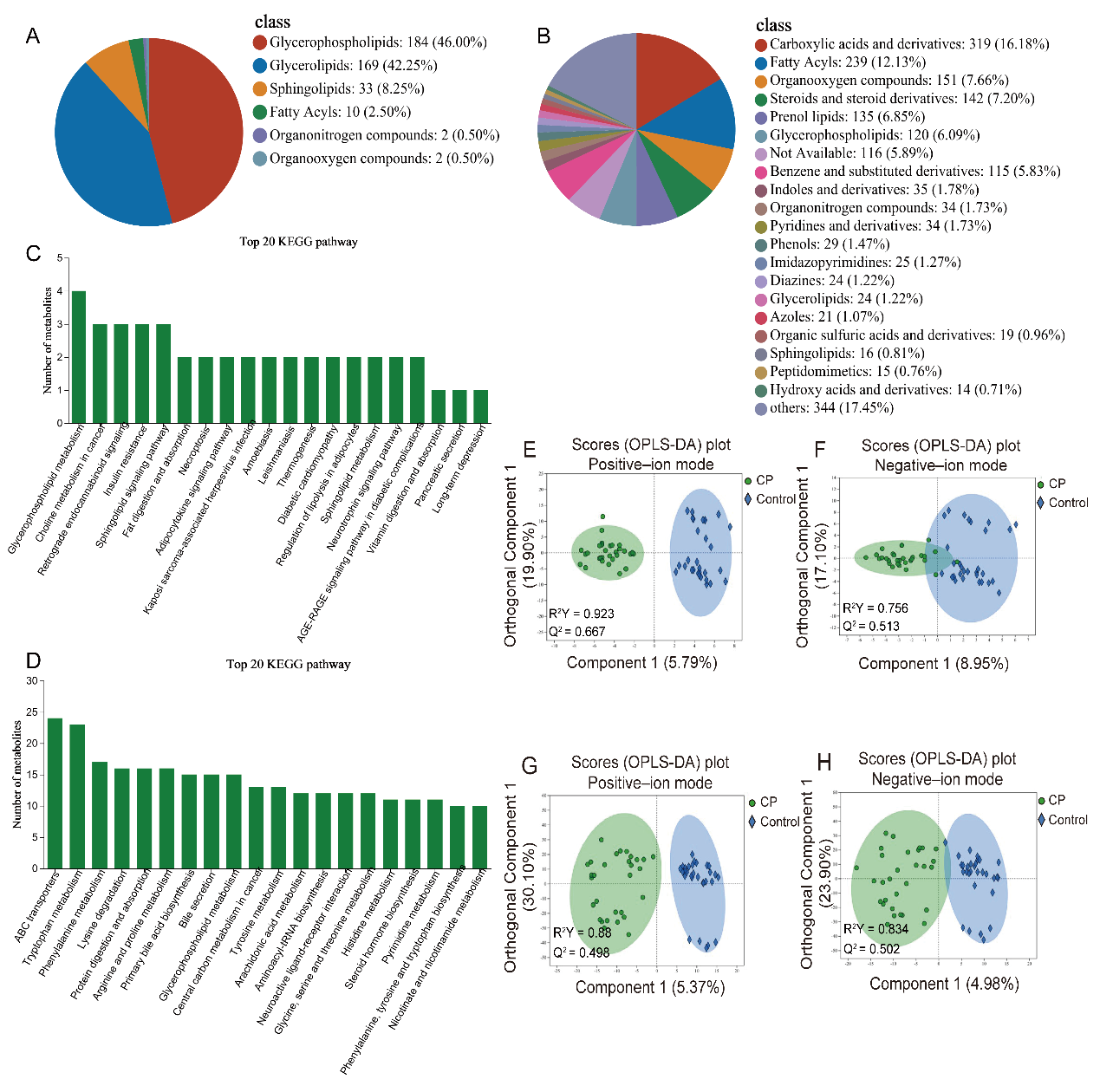


**Figure S4. Annotation, pathway enrichment, and multivariate analysis in the prospective cohort.** (A-B) HMDB compound classification of identified lipids (A) and polar metabolites (B). (C-D) Top 20 KEGG pathways enriched by identified lipids (C) and metabolites (D). Pathways are ranked from left to right by the number of mapped metabolites; higher bars indicate more active pathways. (E-F) OPLS-DA score plots of lipidomics in positive (E) and negative (F) ion modes. (G-H) OPLS-DA score plots of metabolomics in positive (G) and negative (H) ion modes.

**Supplementary Figure 5**


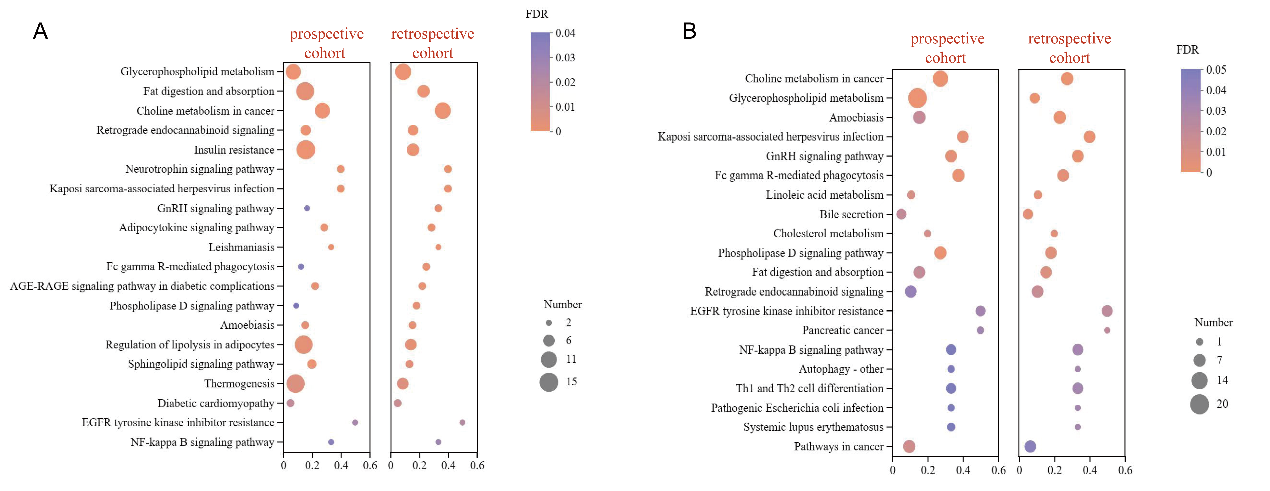


**Figure S5. Joint KEGG pathway enrichment analysis of differential metabolites from prospective and retrospective cohorts.** (A) Bubble plot of lipidomic KEGG pathway enrichment across multiple datasets. (B) Bubble plot of metabolomic KEGG pathway enrichment across multiple datasets. The x-axis represents the enrichment factor, and the y-axis lists KEGG pathways. Bubble size indicates the number of metabolites mapped to each pathway, with larger bubbles representing greater metabolite counts. Bubble color corresponds to enrichment significance, with darker colors indicating lower *P* values.

**Supplementary Figure 6**


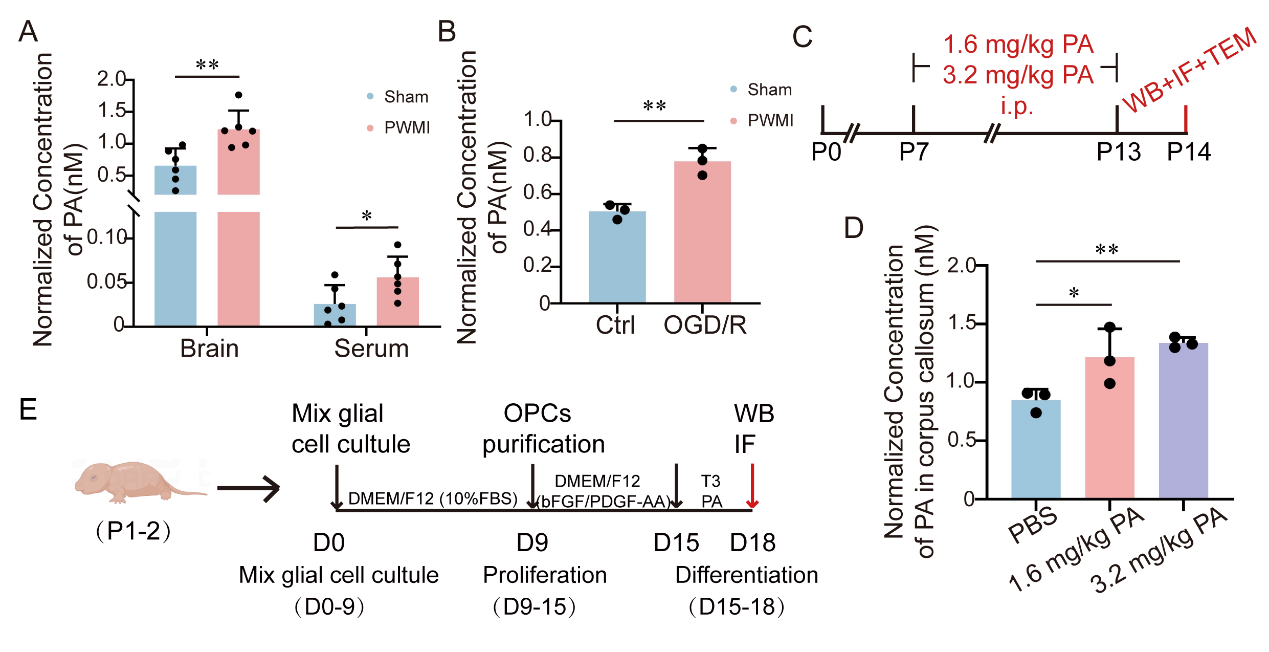


**Figure S6. Experimental design and measurement of PA levels in vivo and in vitro.** (A) Fluorometric assay of PA levels in brain tissue and serum from PWMI mice and the Sham (n = 6). (B) Fluorometric assay of PA levels in primary OPCs from OGD/R (6 h OGD, 24 h reoxygenation) and control groups (n = 3). (C) Schematic diagram of the in vivo experimental design. (D) Fluorometric assay of PA levels in brain tissue from mice administered different PA doses (n = 3). (E) Schematic diagram of the in vitro experimental design. Data are presented as mean ± SD; * *P* < 0.05, ** *P* < 0.01.

**Supplementary Figure 7**


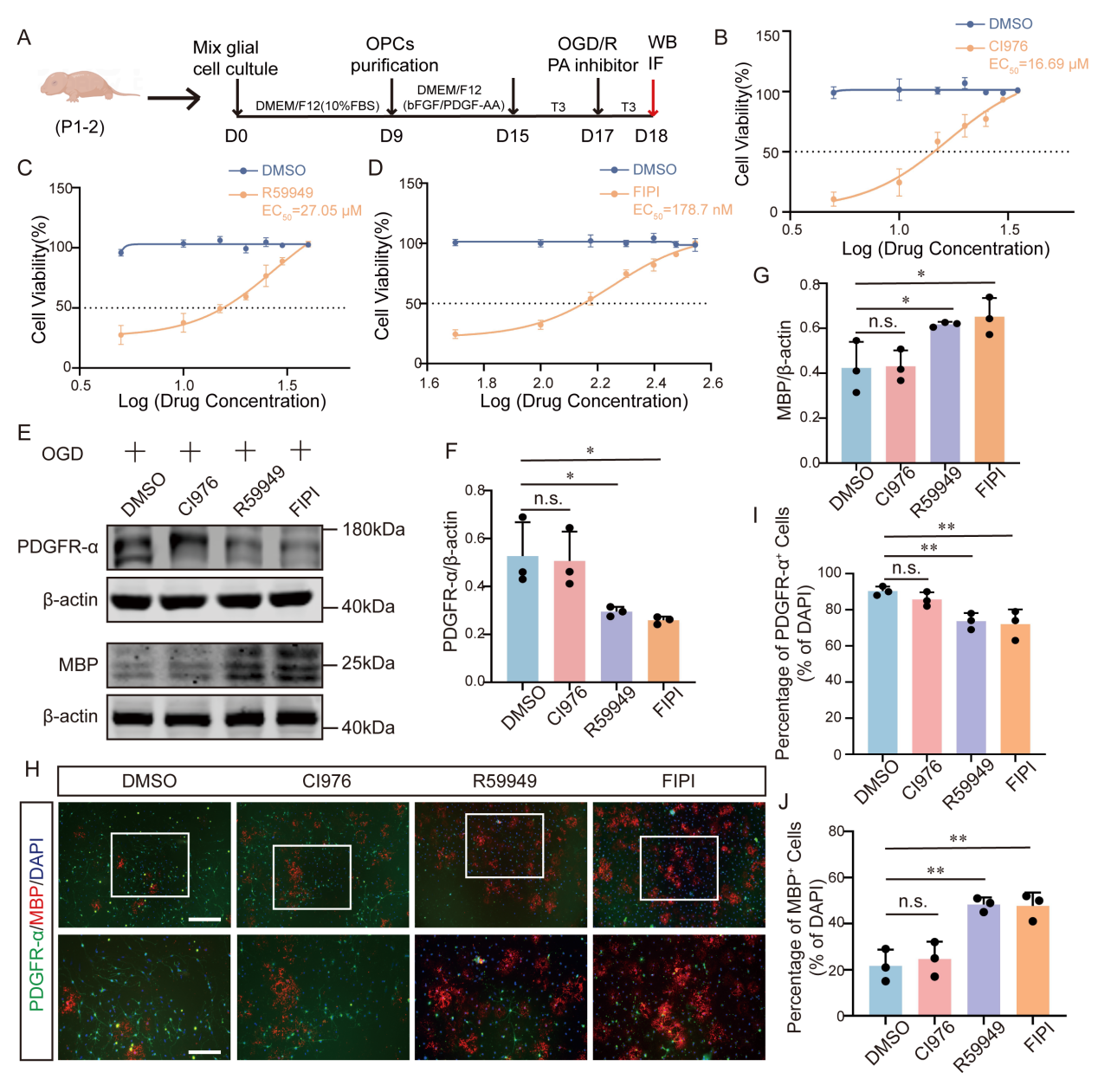


**Figure S7. PA synthesis inhibitors promote OPC differentiation under OGD/R conditions.** (A) Schematic diagram of the in vitro experimental design. (B-D) CCK-8 assays establishing effective concentrations of the PA synthesis inhibitors CI976 (LPAAT inhibitor, 15 µM), R59949 (DGK inhibitor, 25 µM), and FIPI (PLD inhibitor, 150 nM). (E-G) Western blot analysis and quantification of PDGFR-α and MBP expression in OPCs treated with the indicated inhibitors under OGD/R (OGD 6 h, R 24 h) (n = 3). (H-J) Immunofluorescence staining of PDGFR-α⁺ and MBP^+^ cells under the same conditions; high-magnification images correspond to boxed regions in the low-magnification panels (scale bars: low, 200 µm; high, 100 µm; n = 3). Data are presented as mean ± SD; * *P* < 0.05, ** *P* < 0.01.

**Supplementary Figure 8**


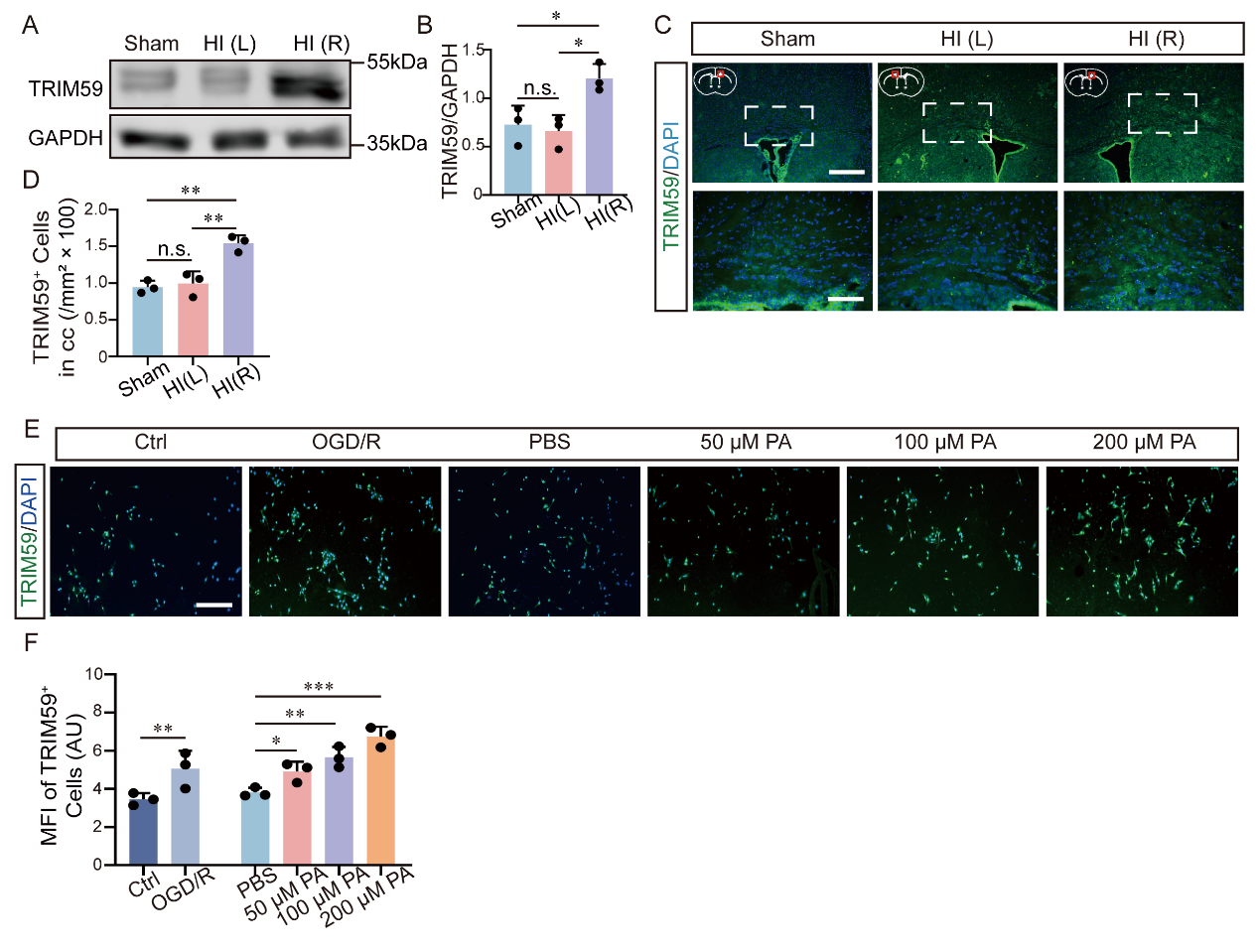


**Figure S8. TRIM59 expression in the corpus callosum and OPCs under PWMI and OGD/R conditions.** (A-B) Western blot analysis of TRIM59 expression in the corpus callosum of PWMI model mice with quantification (n = 3). (C-D) Immunofluorescence staining showing changes in the number of TRIM59⁺ cells in the corpus callosum of PWMI model mice with quantification. Higher-magnification images are enlarged views of the boxed regions in the low-magnification panels. Scale bars: 100 µm (low magnification), 20 µm (high magnification). n = 3. (E-F) Immunofluorescence staining of OLN-93 cells following OGD/R and PA treatment at different concentrations, with quantification of MFI of TRIM59. Scale bar: 100 µm (n = 3). Data are presented as mean ± SD; * *P* < 0.05, ** *P* < 0.01, *** *P* < 0.001.

**Supplementary Figure 9**


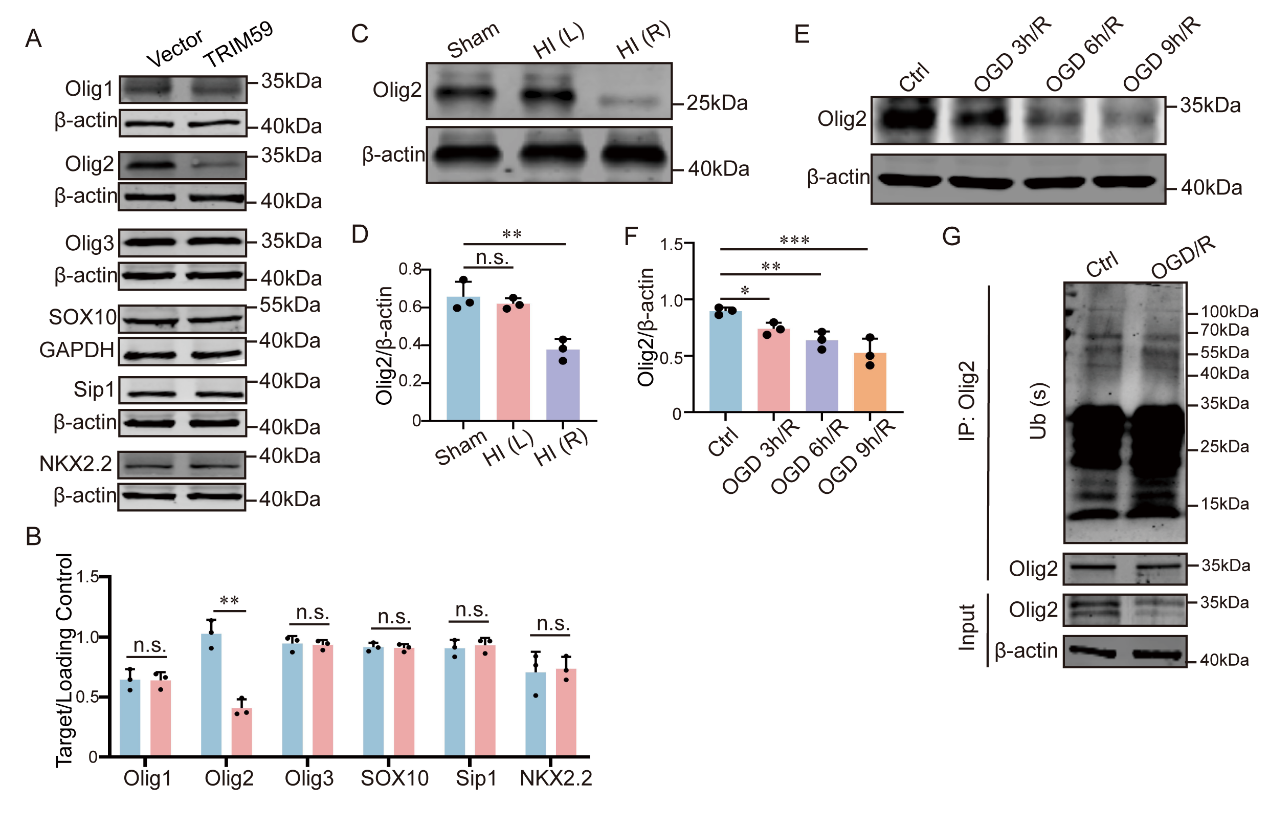


**Figure S9. Increased ubiquitin-dependent degradation of Olig2 in the PWMI model.** (A-B) Western blot analysis showing the effects of TRIM59 overexpression on differentiation-associated transcription factors in OPCs, with quantification (n = 3). (C-D) Western blot analysis and quantification of Olig2 protein levels in the corpus callosum of PWMI mice (n = 3). (E-F) Western blot analysis and quantification of Olig2 protein levels in OPCs exposed to different durations of OGD followed by 24 h reperfusion (n = 3). (G) Co-immunoprecipitation assay showing increased ubiquitination of Olig2 in OPCs under OGD/R conditions. Data are presented as mean ± SD. * *P* < 0.05, ** *P* < 0.01, *** *P* < 0.001.

**Supplementary Figure 10**


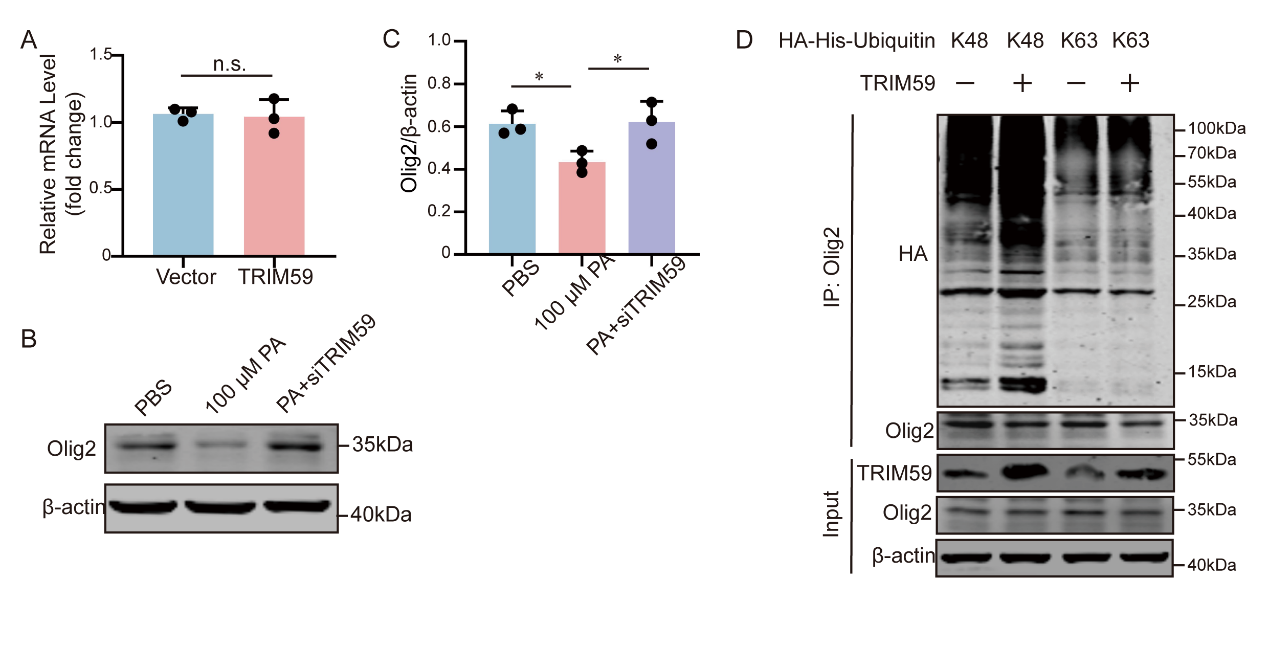


**Figure S10. TRIM59 is required for PA-mediated Olig2 degradation and promotes K48-linked ubiquitination.** (A) RT-qPCR analysis showing that TRIM59 overexpression does not alter Olig2 mRNA levels in OPCs (n = 3). (B-C) Western blot analysis showing that PA treatment reduces Olig2 protein, whereas TRIM59 knockdown (siTRIM59) restores Olig2 expression to near-baseline levels (n = 3). (D) Linkage-specific ubiquitination assay demonstrating that TRIM59 primarily mediates K48-linked polyubiquitination of Olig2. Data are presented as mean ± SD. * *P* < 0.05.

Supplementary Table 1 The sequences of siRNAs using for gene silencing

|  | Sense | Anti-sense |
| --- | --- | --- |
| siTRIM59 | ATGCACAATTTTGAGGAGGAG | TCAACGAGAAACTATTTTCC |

Supplementary Table 2 The primers using for quantitative RT-PCR

| Primer Name | Sequence 5’-3’ |
| --- | --- |
| GAPDH(forward) | GGAGAGTGTTTCCTCGTCCC |
| GAPDH(reverse) | ATGAAGGGGTCGTTGATGGC |
| TRIM59 (forward) | GATGTTGTCACCTGCCCTGA |
| TRIM59 (reverse) | CACACGTTGGCGAACATCAT |
| Olig2(forward) | ATCTTCCTCCAGCACCTCCT |
| Olig2 (reverse) | CCGATGGAGACTTGAGCAGG |

Supplementary Table 3 Multiple linear regression analysis

| **Variates** | **Corrected R^2^** | **B** | **SE** | **β** | **t** | ***P*** |
| --- | --- | --- | --- | --- | --- | --- |
| **PA (18:3/22:6)** | 0.202 | 1.131 | 0.391 | 0.479 | 2.889 | 0.007^**^ |
| **LPC (22:6)** | 0.102 | 1.285 | 0.621 | 0.364 | 2.070 | 0.048^*^ |
| **LPC (20:2)** | 0.102 | 1.089 | 0.526 | 0.364 | 2.070 | 0.048^*^ |
| **TG (18:0/18:0/18:0)** | 0.112 | 0.551 | 0.256 | 0.377 | 2.156 | 0.040^*^ |
| **PA + LPC + TG** | 0.419 | 0.992 | 0.212 | 0.663 | 4.682 | < 0.001^***^ |

B: nonstandardized regression coefficient; SE: standard error; β: standardized regression coefficient; * *P* < 0.05, ** *P* < 0.01, *** *P* < 0.001.
