# Supplementary material for: Phosphatidic Acid‐TRIM59‐Olig2 Signaling Couples Metabolic Dysfunction to Myelination Failure in PWMI
Source: Adv Sci (Weinh). 2026 Feb 18;13(25):e21296. doi: 10.1002/advs.202521296 (PMC13137782; doi:10.1002/advs.202521296)

Figure 5A

PDGFR-α PDGFR-α β-actin


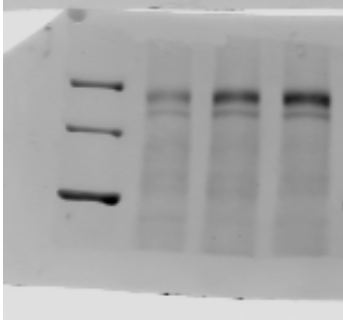

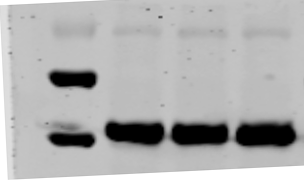


MBP MBP β-actin


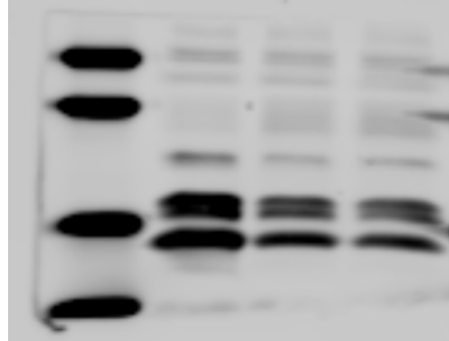

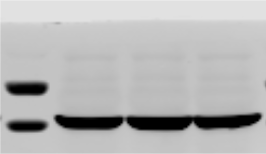


Figure 5I

PDGFR-α PDGFR-α β-actin


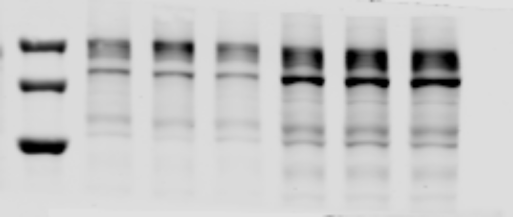

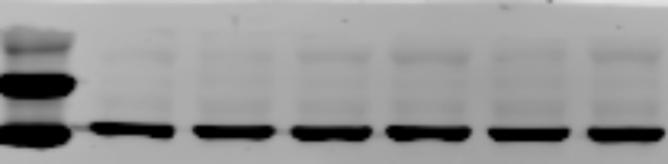


MBP MBP β-actin


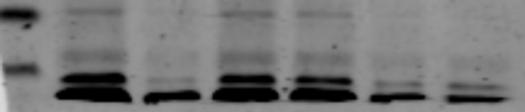

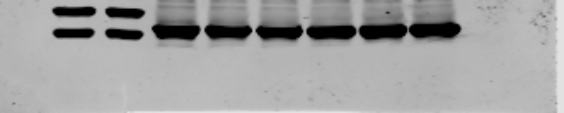


Figure 6G

TRIM59 TRIM59 GAPDH


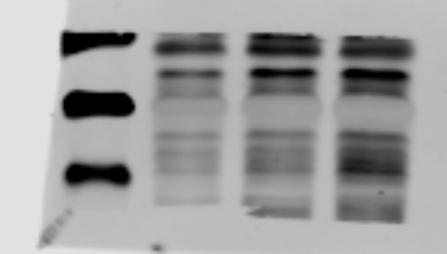

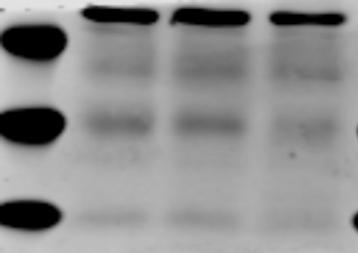


Figure 6I

TRIM59 TRIM59 GAPDH


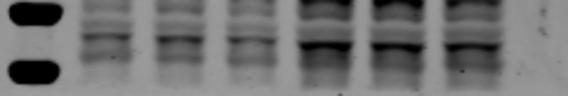

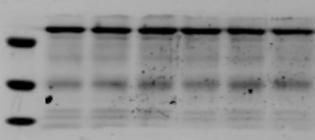


Figure 6L

TRIM59 TRIM59 GAPDH


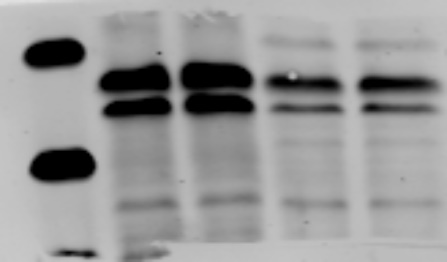

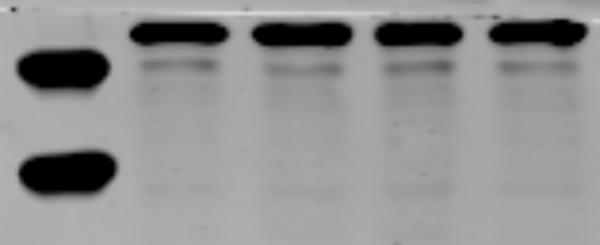


Figure 6N

PBS-TRIM59 PBS-TRIM59 GAPDH


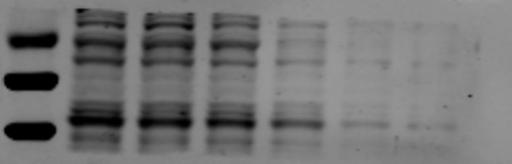

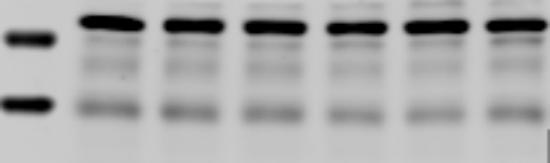


PA-TRIM59 PA-TRIM59 GAPDH


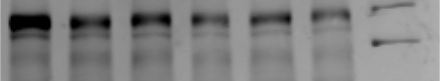

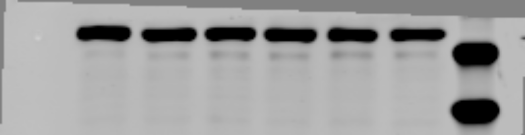


Figure 7A

Olig2 Olig2 β-actin


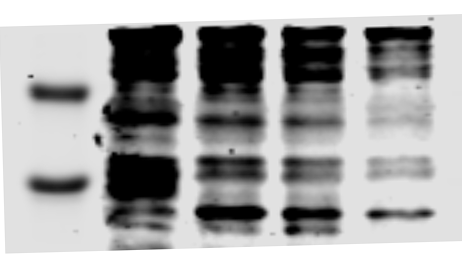

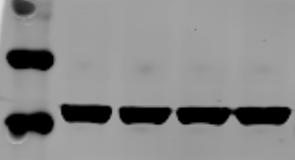


Figure 7D

Ub-Olig2 IP-Olig2


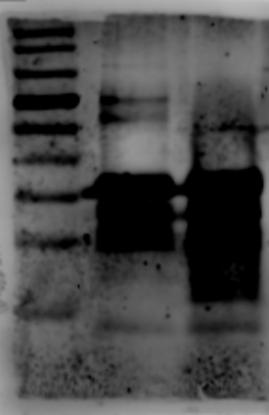

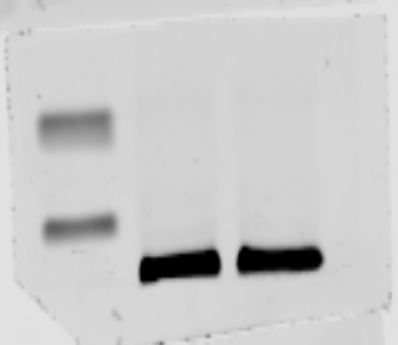


Input Olig2 Input β-actin


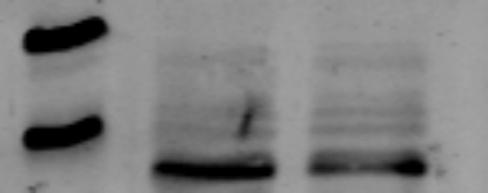

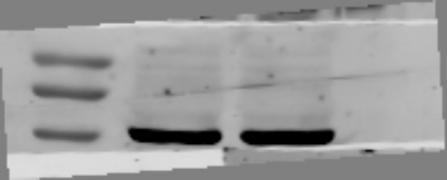


Figure 7E

MG132


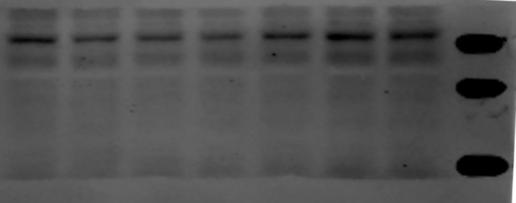

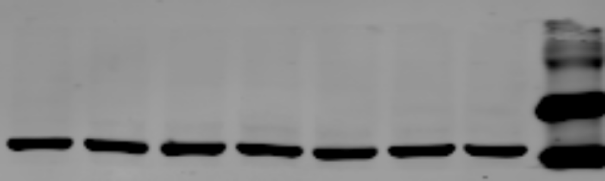


CQ


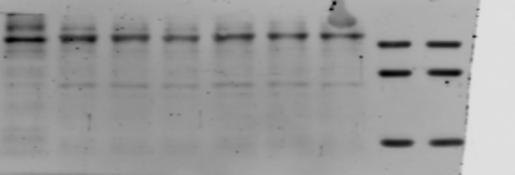

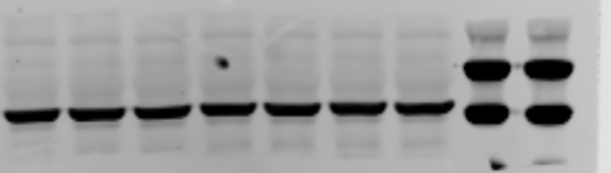


Figure 7F

Olig2 Olig2 β-actin


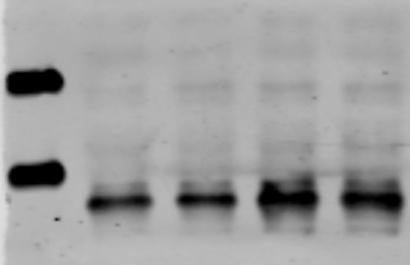

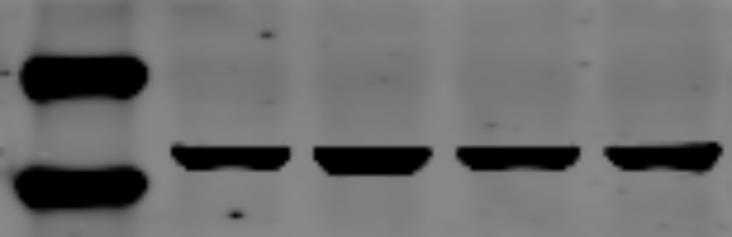


Figure 7H

Ub-Olig2 IP-Olig2


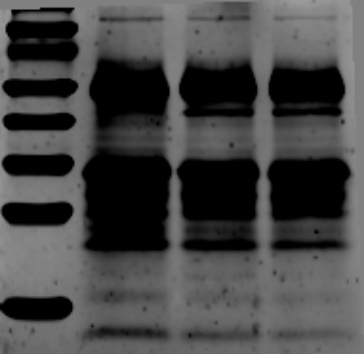

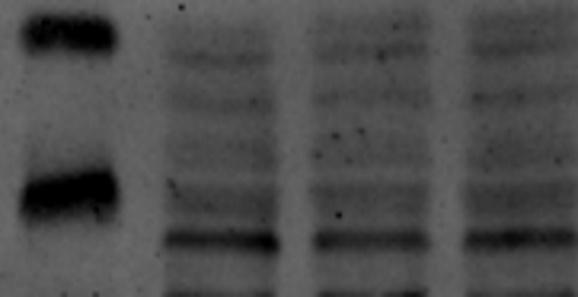


Input Olig2 Input β-actin


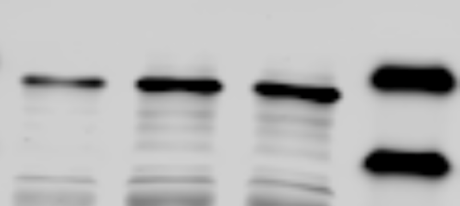

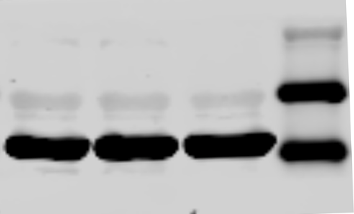


Figure 7I

IP: Olig2-Olig2 IP: Olig2-TRIM59


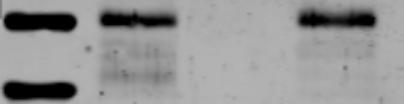

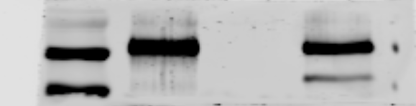


IP: TRIM59-TRIM59 IP: TRIM59-Olig2


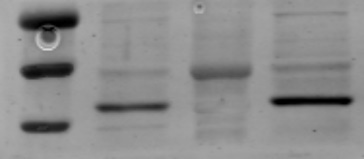

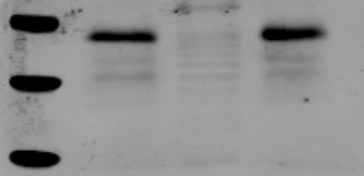


Figure 7L

TRIM59 TRIM59 GAPDH


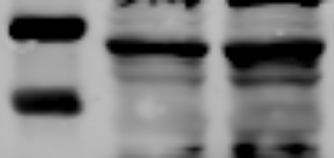

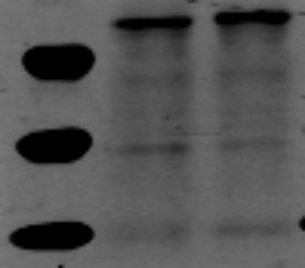


Olig2 Olig2 β-actin


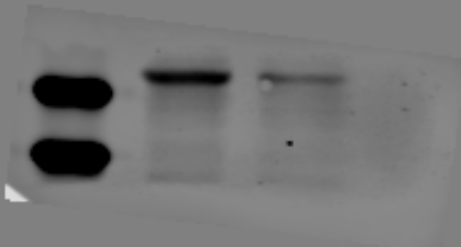

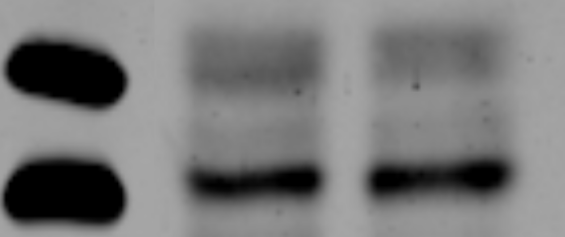


Figure 7N

Ub-Olig2 IP-Olig2


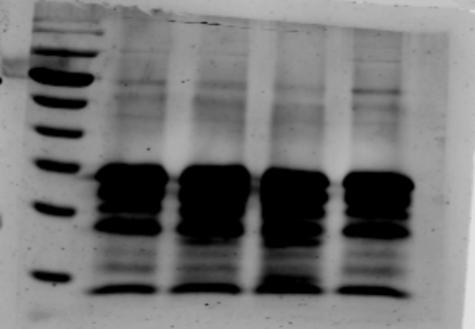

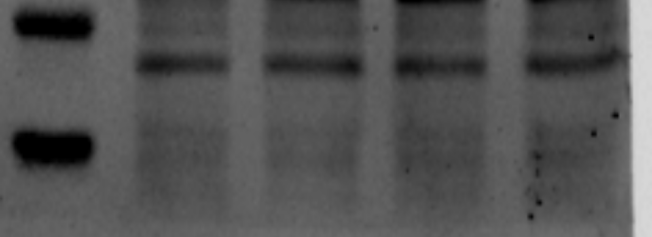


Input Olig2 Input β-actin


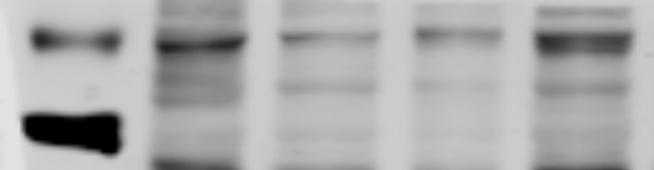

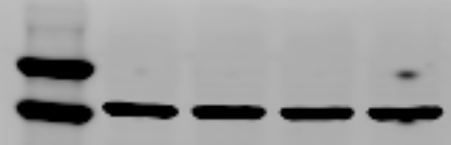


Figure 8C

TRIM59 TRIM59 GAPDH


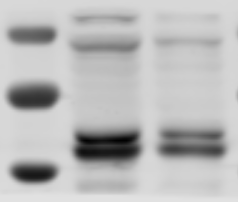

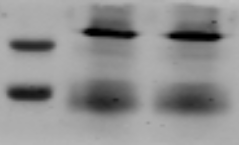


Olig2 Olig2 β-actin


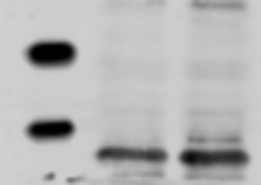

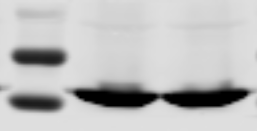


PDGFR-α PDGFR-α β-actin


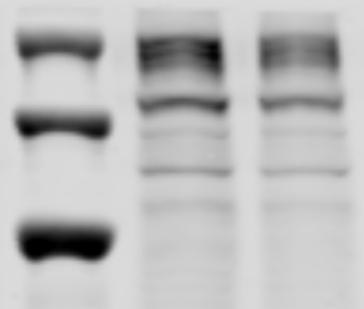

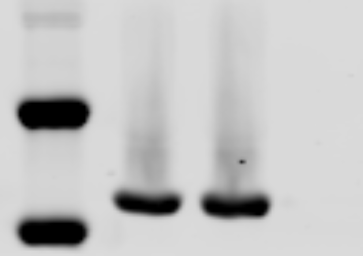


MBP MBP β-actin


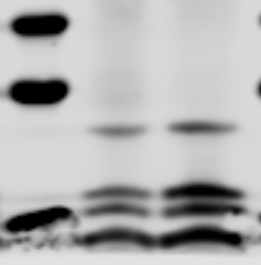

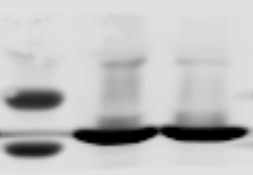


Figure S7E

PDGFR-α PDGFR-α β-actin


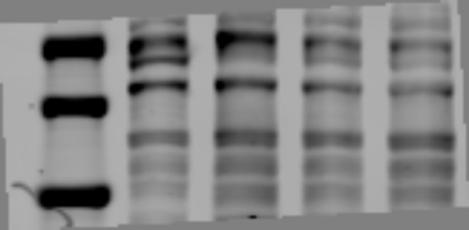

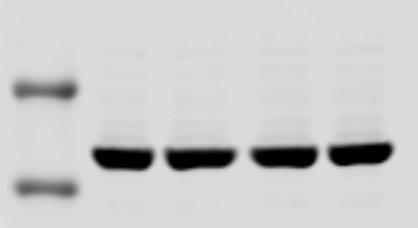


MBP MBP β-actin


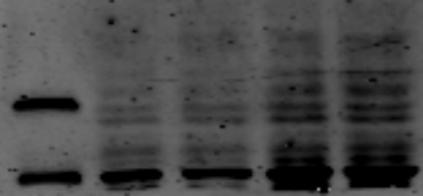

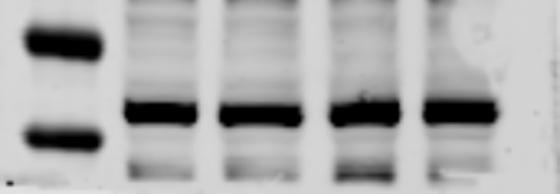


Figure S8A

TRIM59 TRIM59 GAPDH


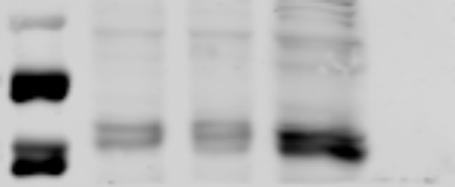

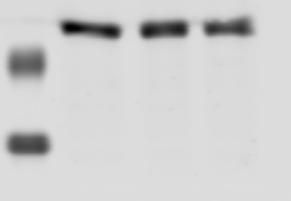


Figure S9A

Olig1 Olig1 β-actin


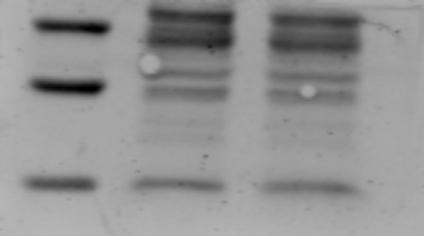

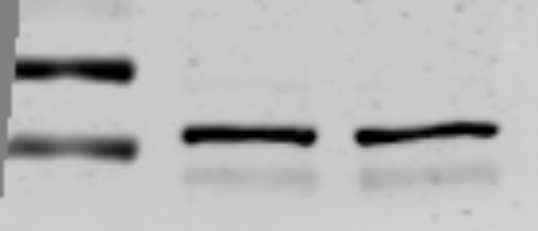


Olig2 Olig2 β-actin


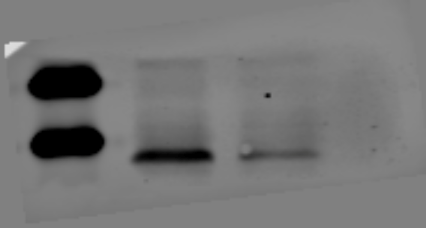

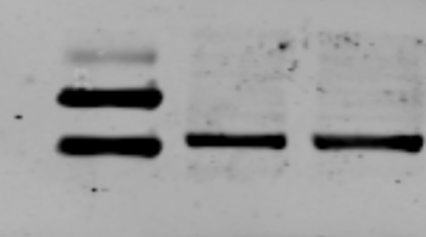


Olig3 Olig3 β-actin


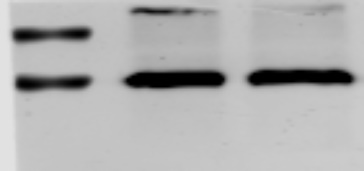

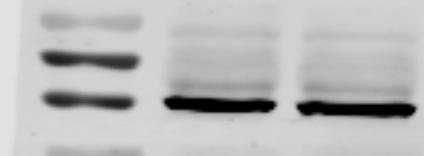


SOX10 SOX10 GAPDH


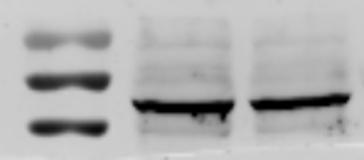

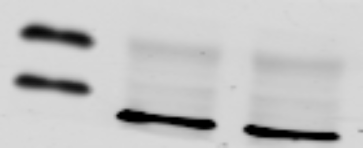


Sip1 Sip1 β-actin


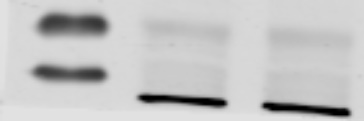

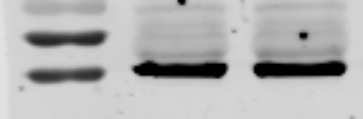


NKX2.2 NKX2.2 β-actin


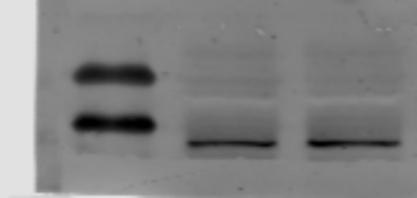

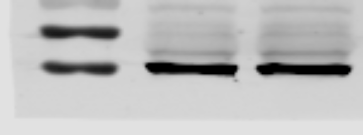


Figure S9C

Olig2 Olig2 β-actin


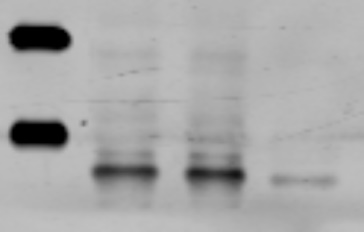

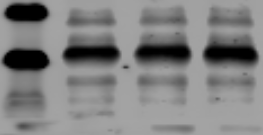


Figure S9E

Olig2 Olig2 β-actin


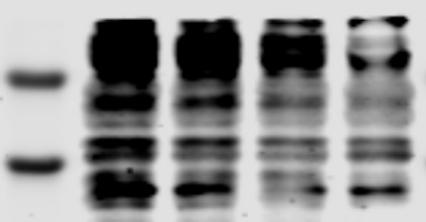

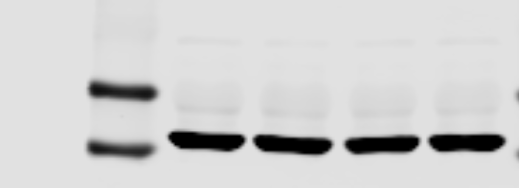


Figure S9G

Ub-Olig2 IP-Olig2


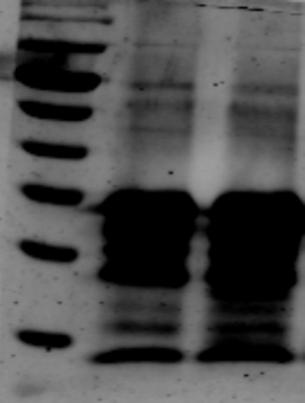

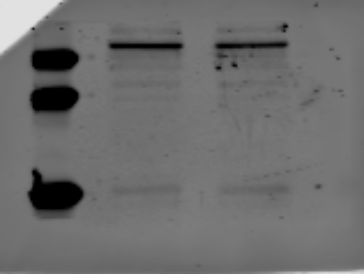


Input Olig2 Input β-actin


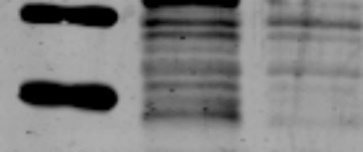

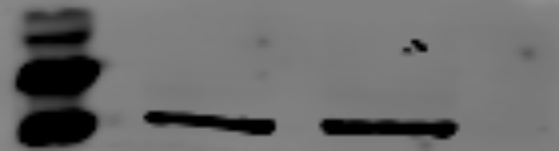


Figure S10B

Olig2 Olig2 β-actin


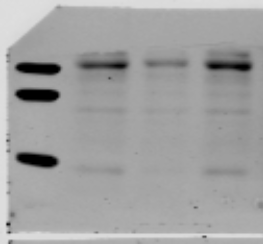

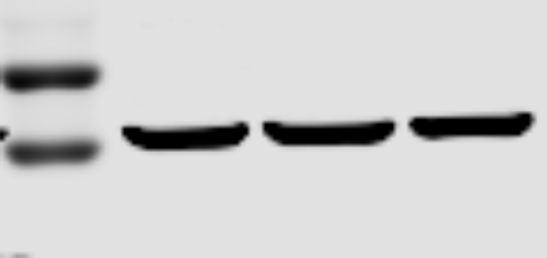


Figure S10D

HA IP-Olig2


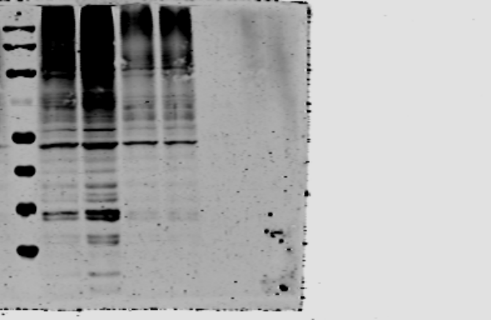

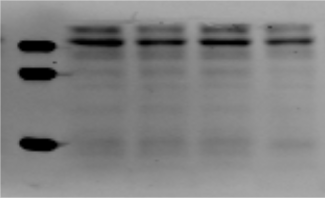


Input-TRIM59 Input-Olig2 Input-β-actin


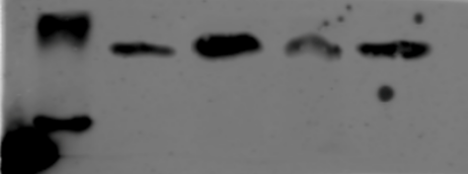

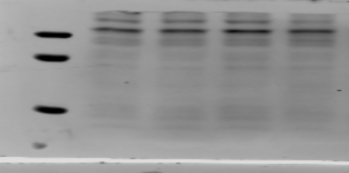

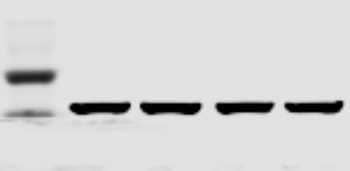

Supplement: Supplementary file 3 — Supporting File 3: advs74495‐sup‐0002‐Data.zip. [file ADVS-13-e21296-s002.zip › advs74495-sup-0002-Data/Full length western blots.docx]
